# Design and Validation of a Sensitive Multisteroid LC-MS/MS Assay for the Routine Clinical Use: One-Step Sample Preparation with Phospholipid Removal and Comparison to Immunoassays

**DOI:** 10.3390/ijms232314691

**Published:** 2022-11-24

**Authors:** Valentin Braun, Hermann Stuppner, Lorenz Risch, Christoph Seger

**Affiliations:** 1 Department of Pharmacognosy, Institute of Pharmacy, Centrum of Chemistry and Biomedicine (CCB), University of Innsbruck, Innrain 80-82, A-6020 Innsbruck, Austria; 2 Dr. Risch Ostschweiz AG, Lagerstrasse 30, 9470 Buchs, Switzerland

**Keywords:** LC-MS/MS, endocrinology, steroid measurement, laboratory medicine

## Abstract

Steroid analysis in clinical laboratories is dominated by immunoassays (IAs) that have a high sample turnover but are inherently limited in trueness, precision, and sensitivity. Liquid chromatography coupled to mass spectrometry (LC-MS/MS) has proved to be a far more capable tool, delivering better sensitivity, specificity, and the possibility of parallel analysis of multiple steroids and metabolites, providing the endocrinologist with more reliable and comprehensive diagnostic information. An LC-MS/MS assay with gradient elution over less than eight minutes and a one-step sample preparation combining protein precipitation with phospholipid removal of off-line solid-phase extraction was developed and validated. It allowed the quantification of 11-deoxycorticosterone (11-DOC), 11-deoxycortisol (11-DF), 17-OH-progesterone (17P), 21-deoxycortisol (21-DF), androstenedione (ANDRO), aldosterone (ALDO), corticosterone (CC), cortisol (CL), cortisone (CN), dehydroepiandrosterone (DHEA), dehydroepiandrosterone sulfate (DHEAS), dihydrotestosterone (DHT), estradiol (E2), progesterone (PROG), and testosterone (TES) in human serum. Interday imprecision was generally better than 15%, trueness was proven by recovery experiments with ISO 17034-certified reference materials, proficiency testing (UK NEQAS), and measuring serum reference standards. In-house comparison against IVD-CE-certified immunoassays (IA) for 17P, ANDRO, CL, DHEAS, E2, PROG, and TES was conducted by assessing leftover routine patient samples and purpose-built patient serum pools. None of the compared routine IAs were meeting the standards of the LC-MS/MS. Insufficient overall comparability was found for ANDRO and 17P (mean bias > +65%). Accuracy limitations at lower concentrations were present in IAs for PROG, E2, and TES.

## 1. Introduction

The analysis of steroidal hormones in body fluids has been one of the mainstays of endocrinological differential diagnosis for decades. A variety of pathologies are associated with shifts in the equilibrium levels of steroidal hormones, such as testosterone, estradiol, cortisol, and aldosterone. Other analytes, often precursor metabolic substances, show elevated concentrations with the onset of certain pathologies [1,2]. Diagnostic work with steroid congeners has changed in the sense that not only new analytical methods have been added, but also that it has been recognized that the metabolic balance of the substances should be considered in its entirety [3]. In this context, it has proven to be extremely advantageous to analyze substance profiles in a joint analytical effort. Since this methodological approach still seems to be denied to the classical ligand-binding assays, the combination of chromatographic substance separation and sensitive/selective quantitative substance detection by mass spectrometers realized in liquid chromatography–tandem mass spectrometry (LC-MS/MS) has taken a new upswing. This is also due to the progress in instrumentation, as more sensitive detectors allow the user to avoid costly and time-consuming concentrating sample preparation procedures such as liquid–liquid extraction (LLE) or solid-phase extraction (SPE).

In this paper, we would like to present a modern multianalyte LC-MS/MS measurement method for the analysis of steroidal hormones in human serum. Using a highly sensitive mass spectrometer, we aimed to design the method so that a maximum sample volume of 100 µL and a simple protein precipitation would be sufficient to quantitatively detect the major steroids (15 analytes). To increase the routine suitability of the established method, the instrumentation, LC-MS/MS, with one-dimensional high-resolution chromatography, electrospray ionization (ESI) in the ion source, and multiple-reaction monitoring (MRM) in the tandem mass spectrometer, was specified. Since the loss of the analyte signal due to the coelution of matrix components, the so-called ion suppression, is a very well-described and known problem in the LC-MS/MS analysis of lipophilic substances [4], great attention was paid to this phenomenon in method development and method evaluation. To ensure the traceability of the platform to the reference methods and/or reference materials, it is essential to use commercial IVD-CE-certified calibrator materials or to make in-house preparations metrologically traceable to these materials. In addition to the generally known parameters such as repeatability and accuracy of the measurement, platform validation in steroid analysis must focus on clarifying possible steroidal and nonsteroidal interferences and evaluating the lower assay detection limits to enable reliable and target-oriented diagnostics.

In the following, the multianalyte measurement platform for the analysis of steroid congeners 11-deoxycorticosterone (11-DOC), 11-deoxycortisol (11-DF), 17α-hydroxyprogesterone (17P), 17β-estradiol (E2), 21-deoxycortisol (21-DF), aldosterone (ALDO), androstenedione (ANDRO), corticosterone (CC), cortisol (CL), cortisone (CN), dehydroepiandrosterone (DHEA), dehydroepiandrosterone sulfate (DHEAS), dihydrotestosterone (DHT), progesterone (PROG), and testosterone (TES) (see Scheme S1 for details) in human serum samples established under the abovementioned conditions will be presented, its validation will be discussed, and the limitations of the method will be pointed out.

## 2. Results

### 2.1. Method Development and Validation

Neutral compound steroids usually have rather poor ionization efficiencies and are therefore inherently challenging to measure by LC-MS/MS. The steroids analyzed in this assay are very different in their detailed structures, which also heavily affects their ionization. Moreover, the dynamic range of the concentrations to be covered is very high (<0.02 to >30,000 nmol/L). As a rich class of endogenous compounds, the risk of interferences or high background in steroid analysis coming from structural analogues such as metabolites or side-products of biosynthesis with the same mass-to-charge ratio (*m*/*z*) is high. Additionally, all possible interferences coming from the complex matrix of the serum must be considered.

Therefore, designing an assay for the fast and accurate routine measurement of multiple different steroids in one single run is a big challenge. The key task in method optimization was to find a good balance in instrument settings and sample preparation to get optimal results. General method characteristics affecting all analytes such as ESI settings or choice of mobile phase were tuned with a primary focus on the most challenging analyte, E2, as it had the weakest ionization efficiency and, at the same time, needed to be measured at the lowest concentrations. For highly abundant analytes with good ionization yields settings with lowered signal yield (“detuning”) were chosen either to avoid detector saturation or to increase the selectivity of the selected ion transition. 

#### 2.1.1. Mass Spectrometry

Ion transitions finally selected for every analyte and internal standard (IS) are listed in Table S1, with the respective optimized instrument parameters for collision energy (CE), declustering potential (DP), and collision cell exit potential (CXP). Entrance potential could be set to 10 V for all ion transitions. Instrument parameters were usually tuned for the highest signal intensity. However, some CE values were detuned to get more selectivity (ANDRO) or better linearity at higher concentrations (PROG, DHEAS).

Ion source parameters are summarized in Table S2. As these parameters cannot be individualized for all ion transitions, ion source conditions were specially optimized for the lower abundant transitions of analytes E2 in negative ionization and DHT and ALDO in positive ionization mode.

The best ionization efficiency for most steroids is usually achieved in the positive mode, as most steroids analyzed in this assay share a 4-ene-3-one group in the A-ring (delta-4 steroids), which facilitates protonation and the formation of an [M + H]^+^ ion [5]. Therefore, steroids such as TES or PROG showed significantly better signal intensities than steroids such as DHEA or DHT, which have only an unconjugated ketone group at the A-ring.

Despite the better ionization in positive mode, CL and CN were chosen to be measured in the negative mode, selecting the [M − CH_2_OH]^−^ ion at the first quadrupole (Q1), as reported previously [6]. CL showed detector saturation with highly concentrated samples in the positive mode. Due to the lower signal intensities using negative ionization, we achieved better linearity while still retaining enough sensitivity to cover the lower ends of the reference ranges, and better selectivity can also be expected [7].

Ammonium fluoride (NH_4_F) was used as an additive to the mobile phases in a concentration of 0.2 mmol/L. It acts as an ionization enhancer, especially in negative ionization mode, but also had enhancing effects or was neutral in positive ionization mode. It was used frequently in recently published steroid LC-MS/MS assays and showed similar enhancing effects of 10 times and was greater in comparison to mobile phases without additives or with the also commonly used additive formic acid [6,8,9,10]. In negative ionization mode, the fluoride anion adduct [M + F]^−^ acts as an intermediate product, which decomposes in-source from the [M − H]^−^ anion to hydrogen fluoride (HF) [11]. In the concentrations used in our method, no damage to the instrument was observed due to the formation of HF during the analysis. However, it was reported in a recent paper that a sensor of the LC instrument was damaged due to HF formation after using a formic acid containing a needle wash solution and NH_4_F containing mobile phases simultaneously [12]. Therefore, we always carefully flushed the instrument with 20% isopropanol before purging with NH_4_F containing mobile phases if other methods were used on the same instrument before. If using NH_4_F as an additive, it is also recommended to use the analytical column exclusively for the assay. Additionally, if changing mobile phases without flushing in-between or using the same column with different methods happened accidentally, the signal-enhancing effect of NH_4_F was significantly reduced, an effect also observed by others [13]. This affected especially lower-abundant analytes such as E2, where the sensitivity of the assay heavily relies on the effects of NH_4_F.

All ion transitions were recorded in the so-called “scheduled MRM” (sMRM) mode. In this mode, a time window and the expected retention time (RT) of the peak, which is in the middle of the time window, is defined for every ion transition. The time window was set to 40 s for all but the DHEAS transitions, where the window was set to 60 s because of a higher peak width and instable RTs due to the ionic nature of the molecule. Dwell times for each transition and the overall cycle time at each time point of an analysis run was estimated and optimized using a tool included in the Analyst software (Sciex sMRM calculator). Dwell times and graphical depictions of the final sMRM transitions are shown in Figure S1 and Table S3. It was not possible to exactly determine the dwell times or cycle times, as there is no open-source description of the algorithm used by the manufacturer to calculate the dwell times based on the used settings, so the results calculated by the tool provided by Sciex is the best estimate we could get. Nonetheless, dwell times and data points per peak were higher using sMRM instead of the classic MRM mode in which all transitions are recorded over the whole run. In classic MRM mode, the dwell times would have had to be set to 10 to 15 ms per transition to get just enough data points per peak. In sMRM mode, however, ≥10 data points per peak were reached and the estimated dwell times were at least at 21 ms per transition.

Signal summing of the same ion transition—also called transition summing—was applied to the E2 quantifier (QN) transition by recording it five times. This led to an equivalent increase in the absolute signal and a better signal-to-noise ratio (see Figure S2), enhancing particularly the lower limit of quantification for E2. This was also described previously in a method specifically designed for estrogens [14]. Increasing the dwell time instead enhanced only the quality of the single data points, as more time was spent on signal averaging. However, if the dwell time was high enough for each measurement, only summing multiple ion traces together had a significant effect on the sensitivity. Combining transition summing with sMRM allowed us to use this technique even in a crowded 15-analyte method and guaranteed enough dwell time for every transition. Whether or not the summing of transitions also had a beneficial effect on overall precision (also at higher concentrations) could not be determined. At least in a study with immunosuppressant drug everolimus, transition summing only enhanced precision at lower concentrations, leading to lower quantification limits, but it had no significant effect on overall precision [15].

Transition summing was also applied to DHT, as we struggled to achieve the aimed sensitivity. However, the effect was not nearly as significant as observed with E2. Contrary to E2, the DHT ion transition had a rather high baseline signal of up to 10^5^ counts per second (cps) and, consequently, a low signal-to-noise ratio. Transition summing seems to follow the principle “bad input–bad output”. Transition summing is nonselective, also enhancing possible interferences and background signals, which is probably why it had little effect with DHT.

#### 2.1.2. Liquid Chromatography

**Figure 1:**
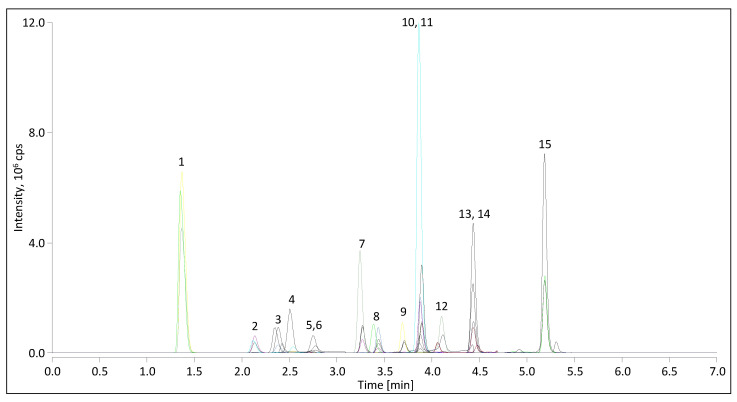
Overlay chromatogram of all monitored ion transitions of a QC level II sample of 11-deoxycorticosterone (11-DOC), 11-deoxycortisol (11-DF), 17-OH-progesterone (17P), 21-deoxycortisol (21-DF), androstenedione (ANDRO), aldosterone (ALDO), corticosterone (CC), cortisol (CL), cortisone (CN), dehydroepiandrosterone (DHEA), dehydroepiandrosterone sulfate (DHEAS), dihydrotestosterone (DHT), estradiol (E2), progesterone (PROG), and testosterone (TES). Each transition was only monitored at the respective time window. All three transitions (QN, QL, IS) are depicted for every analyte. Each transition is depicted in an individual color. Peak labeling: 1. DHEAS; 2. CL; 3. CN; 4. 21-DF; 5., 6. E2 and ALDO; 7. 11-DF; 8. CC; 9. DHEA; 10., 11. TES and 17P; 12. DHT; 13., 14. ANDRO and 11-DOC; 15. PROG.

An exemplary chromatogram of a quality control (QC) level II sample with ion transitions of all analytes overlayed is illustrated in Figure 1.

Single chromatograms of the QN ion transition from a mobile phase blank, a zero calibrator, the lowest calibration point, and a serum sample near the lower quantification limit are depicted in Figures S3–S5, together with the RTs and the found concentrations for each analyte. Using a biphenyl column and a water–methanol gradient elution program, baseline separation of all isobaric analytes was achieved in less than 5.5 min, including CN and ALDO, 21-DF, 11-DF, and CC, DHEA and TES, and 17P and 11-DOC. 17P and TES, and 11-DOC and ANDRO, were not separated chromatographically but were easily differentiated in the MS/MS analysis by their different *m*/*z* ratios. Some known steroidal substances were also investigated as interferences and chromatography was optimized respectively (see Section 2.2.6).

Chromatography was not only optimized to separate isobaric analytes and some known interferences, but also to enhance selectivity against visible interference signals of unknown origin. For example, the 11-DOC signal was affected heavily by an interfering signal present in every sample (partly visible in Figure S5 at the end of each 11-DOC chromatogram). Separation of the unknown interferent could be influenced especially by changing the column temperature. While the interferent eluted earlier than 11-DOC at temperatures <35 °C, it eluted later at higher temperatures >45 °C.

Initially, an LC-MS/MS method with a shorter elution time using a steeper gradient (50% B to 100% B in 4 min) and with an oven temperature of only 35 °C was used. Isobaric analytes were well separated, and the method showed good performance in calibrator and QC samples. However, as illustrated in Figure S6, coeluting phospholipids caused major analytical problems in authentic human serum samples due to ion suppression, which affected analytes with RTs between 3.5 min and 4 min. As with some unknown interferences described above, the chromatographic retention of phospholipids was affected differently than that of steroid analytes when changing the column temperature. By increasing the temperature, phospholipids eluted later in relation to the steroid analytes leave less analytes potentially affected. Higher temperatures led to signals and lower analysis time, and, finally, a column oven temperature of 50 °C was chosen as an ideal compromise between assay selectivity and analysis time. Thanks to the accelerated elution at higher temperatures, the gradient could be flattened to the final conditions, which had much less influence on the relative RTs of steroids and phospholipids. However, a flatter gradient further enhanced the selectivity of the method against some unknown interfering peaks.

#### 2.1.3. Sample Preparation

A major design goal in method development was a simple and fast sample preparation with only one major working step. Hence, initially, an LC-MS/MS method was developed relying only on protein precipitation by a methanolic zinc sulfate (ZnSO_4_) solution (62 mmol/L ZnSO_4_ in 80% methanol) in the sample preparation. However, as mentioned above, strong matrix effects in the form of ion suppression were discovered during a prevalidation experiment of that method prototype (see Figure S6). The mean IS peak area of 17P, TES, 11-DOC, and ANDRO in the serum samples was between 50 and 75% smaller than in the QC sample, and the IS of DHT was almost undetectable in the serum samples. The other analytes eluting before 17P or after ANDRO showed an IS peak area loss of only around 25%. Consequently, a modified sample preparation scheme was needed.

The most common source of the matrix effects causing ion suppression are coeluting phospholipids. Therefore, the addition of a phospholipid-removing step in the sample preparation was investigated. Two different phospholipid-removing technologies—HybridSPE (Supelco) and Phree (Phenomenex)—were tested. According to the manufacturer of HybridSPE, their technology works based on strong Lewis acid–base interactions of the electron-donating phosphate moiety of phospholipids with electron-accepting zirconia atoms which are coated on the silica solid phase [16]. The principle of the Phree columns was not published by the manufacturer. Signal output with Phree cartridges was very low for most analytes, independent of the precipitating agent, and since the working principle is not known, no further experiments were conducted with this product. It had to be assumed that steroids were retained in the filter layer of the Phree cartridges. For all further experiments, only the HybridSPE cartridges were used.

Phospholipid removal by HybridSPE technology effectively reduced ion suppression. As illustrated in Figure S6, ion yield was clearly enhanced after phospholipid extraction for all ISs. Except for DHT, an IS peak area recovery between 80 and 100% was achieved after phospholipid reduction. This experiment was conducted with the initially developed LC-MS/MS method. The final method had different chromatography settings, which further optimized the DHT recovery (see Section 2.1.2). The effects of phospholipids on the ion suppression were studied also by postcolumn infusion experiments, in which the efficiency of the phospholipid removal by HybridSPE was assessed by monitoring specific ion transitions of common serum phospholipids (see Section 2.2.5).

Besides its effects on ion suppression caused by phospholipids, it was investigated if the choice of protein precipitation agent had an influence on the extraction recovery (ER) of the steroid analytes in conjunction with the HybridSPE technology. The manufacturer of the HybridSPE cartridges recommends either 1% formic acid in acetonitrile (*v*/*v*) or 1% ammonium formate (*m*/*v*) in methanol or 0.5% citric acid in acetonitrile (*m*/*v*), each in a 1:3 volume ratio of sample-to-precipitation-agent, with a maximum sample volume of 100 µL (serum) for optimal analyte ER [17]. Each recommended precipitation agent was tested, but they were incompatible with the already-established chromatography. The higher organic content led to peak broadening and acid additives potentially interacted with NH_4_F, reducing its signal-enhancing effect.

A methanolic ZnSO_4_ solution was selected as precipitating agent independently before the need of phospholipid reduction was evident. Even though it is not recommended by the manufacturer, using a ZnSO_4_ solution in combination with HybridSPE in the sample preparation for steroid analysis from serum proved to be an effective combination. Results for the ER from the pre- and postspike experiments were >80% for all analytes (see Table S10). Especially the recovery rate of ALDO was impressive, since this analyte showed a high interaction potential with the zirconia atoms in the filter layer in a previous study due to its two carbonyl functions in positions 18 and 20 [18]. In that study, the best recovery for ALDO by the means of HybridSPE was ultimately achieved using the citric-acid-containing precipitation agent mentioned above. The sulfate ions in our method act in a similar way as citrate ions, since both are good electron donors (Lewis bases), inhibiting the binding of chelating compounds such as ALDO (weak Lewis base) with zirconia atoms [19]. Nevertheless, the retention of phospholipids is not affected, since the interaction potential of the phosphate ions with zirconia atoms is higher than that of sulfate ions [20].

The use of a vacuum manifold is recommended by the HybridSPE manufacturer for enabling the filtration process [16]. Since such a device was not available in our laboratory, we tried to use centrifugal force to elute the sample extract through the filter layer. The cartridges were centrifuged at 100× *g* for 3 min and at 500× *g* for 1 min. In the first step, the minimal force for almost full elution was chosen for a similar time as recommended for using the vacuum manifold. The second centrifugation step was necessary for ensuring full elution of all cartridges. Neither the manufacturer nor any report in the literature was found using centrifugation for elution with the HybridSPE technology. A clear eluate and complete passthrough was obtained each time, showing that centrifugation is a good alternative to applying a vacuum if no such device is available.

### 2.2. Validation

#### 2.2.1. Calibration, Regression Model and Linearity

**Figure 2:**
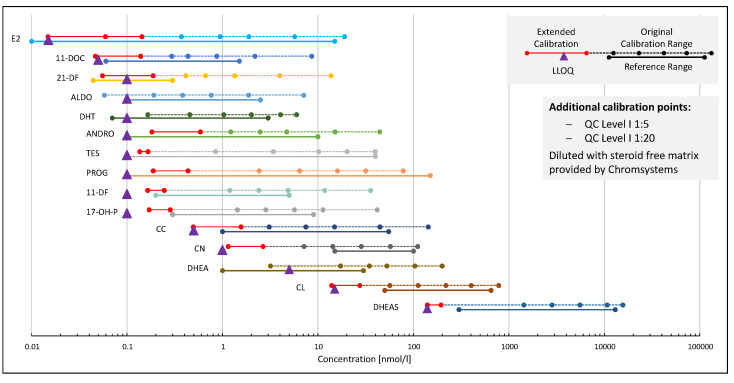
Overview of the concentrations of LLOQs, calibration ranges (original and extended calibration), and human biological reference intervals of 11-deoxycorticosterone (11-DOC), 11-deoxycortisol (11-DF), 17-OH-progesterone (17P), 21-deoxycortisol (21-DF), androstenedione (ANDRO), aldosterone (ALDO), corticosterone (CC), cortisol (CL), cortisone (CN), dehydroepiandrosterone (DHEA), dehydroepiandrosterone sulfate (DHEAS), dihydrotestosterone (DHT), estradiol (E2), progesterone (PROG), and testosterone (TES) on a decadic logarithmic scale. Reference ranges (full lines) and original calibration ranges (dashed lines) as provided by the calibrator manufacturer are color-coded for each analyte. Dots indicate the individual calibrator sample. Extensions of calibration ranges to meet the requirements of the reference range are presented in red, red dots mark the additional calibration samples derived from quality control materials. Violet triangles mark the set lower limit of quantification.

Commercially available materials were used for calibration. Target concentrations were assigned by the manufacturer. Additional calibration points generated by diluting QC materials had to be added at the lower end of the calibration function compared to the out-of-the-box calibration to take advantage of the full sensitivity range of the newly developed assay (see Figure 2). Only with the additional calibration points could the required precision and accuracy in this concentration range be ensured. Calibration curves were constructed from the ratio of analyte to the IS peak area or height (ALDO, DHT). The best-fit regression model was selected as described previously, including comparing residual and bias plots and bias sums between linear and quadratic regression [21,22,23]. Quadratic regression was applied to 17P, DHEAS, CL, and PROG. For all other analytes, a linear regression was sufficient. An F-test comparing the variance at the highest calibrator with the lowest calibrator showed that each analyte has a heteroscedastic data set, which means that the variance is not constant across the calibration range. Therefore, a weighting had to be applied to the regression, providing a more accurate quantification at the lower end of the calibration range. A 1/x^2^ weighting proved to be the best model for each analyte. It was selected based on variance plots in which a linear curve indicates 1/x and a parabolic function 1/x^2^ weighting [21]. The selected regression model and weighting factors, as well as the mean regression parameters and coefficient of correlation (R^2^) obtained from validation experiments, are listed in Table S4. Linearity was excellent over the whole range of every analyte. Mean coefficient of correlation was >0.997 for each analyte. All measurements of samples build by mixing the highest and lowest calibrators stepwise had a bias and accuracy of <±15%. The slope was stable for each analyte over multiple days, with a CV < 5% for most analytes. Only 21-DF showed a higher slope variation with 8.5%. This is in good accordance with other findings in this study, as 21-DF also showed higher CV in QC materials, which both could be caused by the high matrix effects found in the pre- and postspike experiments.

#### 2.2.2. Sensitivity and Limits of Quantification

As a parameter for the sensitivity of the assay, the lower limit of quantification (LLOQ) was assessed for both the QN and the qualifier (QL) transitions. Technically, the LLOQ was defined as the concentration at which the measurement repeatability reached a CV of 20%. Further criteria that had to be considered practically were accuracy, calibration range, signal-to-noise ratio, peak shape, and variable sensitivity due to matrix effects, column lifetime, or variable day-to-day instrument performance. Based on the results of the technical evaluation of the assay and considering biological human serum reference ranges and medical decision points, reasonable validation goals for LLOQs were defined for each analyte. Accordingly, three dilutions of QC level I (LLOQ QC) with concentrations at or near the predefined LLOQ goals were included in the interday experiments for the final confirmation of the LLOQs under near-realistic conditions. Accuracy and imprecision results for LLOQ QCs are summarized together with their respective concentrations in Table 1.

Based on these results and on the criteria mentioned above, the LLOQs were set for each analyte as summarized in Table S5. The LLOQs ranged from 15 pmol/L for E2 to 140 nmol/L for DHEAS. This would allow for quantification below or at the lower end of all biological reference ranges found in human serum for most analytes (Figure 2).

According to the validation results for LLOQ QCs, LLOQs for 21-DF, DHT, DHEA, and ALDO had to be set to slightly higher concentrations than that of the lowest calibrator and the assay could not cover all reference ranges for these analytes. However, despite not covering the whole reference range, the sensitivity of the assay for 21-DF and DHEA can be considered sufficient, because the samples with concentrations above the reference intervals are usually of clinical interest and upper cut-off values (<0.3 nmol/L and <40 nmol/L for 21-DF and DHEA, respectively) are easily covered by the assay [24]. For DHT, male reference ranges are covered well with the found LLOQ of 0.3 nmol/L, and since the upper limit of the female range is covered as well, samples of women with elevated DHT levels could be identified [25]. Upper limits of quantification were set to the highest used calibrators. For analytes 17P and PROG, the samples above the highest calibrator (about 200 nmol/L and 100 nmol/L, respectively) were frequently measured in the proficiency testing scheme UK NEQAS (see Section 2.2.4). As the bias results were in accordance repeatedly, it can be assumed that extrapolation for these analytes is possible.

#### 2.2.3. Imprecision

Imprecision of the assay was assessed by repeated measurements of QC materials and pooled human serum samples on five different days, and by calculation of CVs on an interday and intraday basis thereof. The validation goal was to stay within a CV of 15% at all samples and at 20% at samples near LLOQ. For each analyte and sample, the calculated CVs for QC samples are listed in Table 1, and for pooled human serum samples, they are listed in Table 2.

Intra-assay imprecision at QC levels I–III was below 7% for all analytes, whereas only analytes DHEA, 21-DF, ALDO, DHT, and CN showed values above 4%. In human serum, intra-assay imprecision was similar with CV values below 9% for all analytes at concentrations above LLOQ. For 21-DF, no imprecision assessment in human serum samples was possible because all found concentrations were below the LLOQ of 0.1 nmol/L. Interassay imprecision was found to be below 15% at all QC levels. For CN, ALDO, DHT, and E2 in the found CVs ranged between 5% and 10% and for 21-DF, the average CV values were around 10% for all QC levels. For all other analytes, CVs better than 5% were calculated at all QC levels. Similar results were found in human serum samples. The assay delivered reproducible results over five days, reaching CVs below 15% for each analyte, except for two 11-DOC samples with near LLOQ concentrations measured with a precision from 15 to 20%. Results for precision of LLOQ QCs are discussed in Section 2.2.2.

#### 2.2.4. Accuracy

Accuracy was estimated on three levels of evidence. Best evidence was achieved by analyzing the reference materials, followed by taking part in the UK NEQAS proficiency testing scheme, and basic evidence was provided by recovery experiments and measuring the internal QC materials with every run.

Generally speaking, the best accuracy assessment is achieved by measuring the matrix-matched secondary higher-order reference materials. For TES, E2, CL, and PROG, the certified reference materials (CRMs) of human origin were available at multiple analyte levels. These materials were measured in singleton with every batch on five different days. Accuracy was excellent for TES, with a mean bias of −0.1% and +2.4% against target values and good for E2 ranging from +0.4% to +6.9% at three levels (Table S6). For CL (+7.5% and +6.7%) and PROG (+11.5% and +11.6%), a significantly higher positive bias was observed. Although analytically significant, found bias for PROG and CL is still within the allowable limits and therefore can be considered acceptable for clinical use, also because precision is very good over the whole reference range (see Section 2.2.3). We were not able to reproduce the CL and PROG bias in QC materials, and it remained present after changing the calibration material lot. Hence, it seems possible that the preparation of calibrators and QC materials by the manufacturer might be the cause of this issue.

Taking part in the proficiency testing scheme of UK NEQAS for steroids over multiple distributions (n = 24) revealed good to very good accordance with other LC-MS methods. The main overall results are summarized in Table S7 and the Bland and Altman plots illustrating differences against target values for all individual measurements are shown in Figure S7. Average bias compared to the LC-MS peer group mean was below ±5% for all parameters, but PROG revealed a more significant positive bias of +9.8%. The method performed well when looking at the variability of the found bias values, with standard deviations (SD) below 10% for all parameters except ALDO. While the mean bias for ALDO was very good with −2.3%, the SD of bias was 17.6%, as there were a lot of results with a bias >±15%.

**Figure 3:**
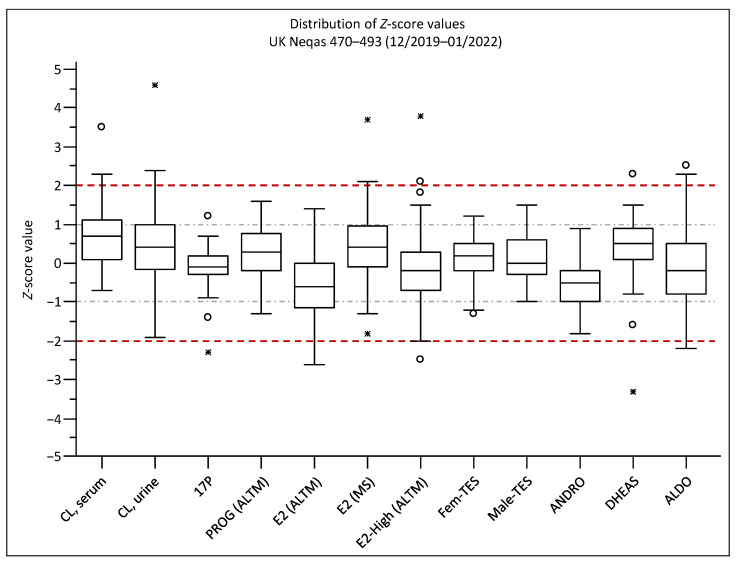
Boxplots showing distribution of *Z*-score values for included parameters of the UK NEQAS scheme for steroids. 1*Z* = one SD of the group mean. LC-MS group mean as official target for 17-OH-progesterone (17P), androstenedione (ANDRO), aldosterone (ALDO), cortisol (CL), dehydroepiandrosterone sulfate (DHEAS), estradiol (E2), progesterone (PROG), and testosterone (TES). TES testing is split up in a scheme for samples from females (Fem-TES) and males (Male-TES). E2 testing is split up in regular samples (E2) and samples from E2 substitution (E2-High). All-methods-trimmed mean (ALTM) as official target for PROG and E2. LC-MS group mean (MS) available for E2. Too few LC-MS participants for LC-MS group statistics for PROG and E2-High. Sample numbers per analyte ranged from 52 to 80 (see Table S7 for details). The box of the boxplot ranges from the 1st to the 3rd quartile (interquartile range (IQR), 25th to 75th percentile), with the horizontal line indicating the median (50th percentile). Whiskers are positioned at the highest/lowest data point within the ±1.5-fold distance of the IQR. Circles indicate data points in the value range between the ±1.5-fold and ±3.0-fold IQR, stars indicate data points exceeding the ±3.0-fold IQR.

Bias results of every single sample were compared to the SD of the according target value by calculating the *Z*-score values. They give a better estimate of how our method performed in comparison to the LC-MS peer group, as it better considers the uncertainty of the target values. Most results were within 1*Z* (one SD) of the target, showing also very good accordance of the newly developed method with the other LC-MS results. The distribution of the *Z*-values is depicted in Figure 3 and the average *Z*-values in Table S7.

In this proficiency testing scheme, target values are usually the LC-MS group mean and testing materials are based on pooled human serum samples. This means that the quality of the feedback provided by this scheme on the quality of the own-assays results is very high and the target values are a good representation of the true value. It was one of the key experiments for the development of this assay. Problems with accuracy for TES revealed in this scheme led to the discovery of strong matrix effects caused by phospholipids, as discussed in Section 2.1.2, Section 2.1.3 and Section 2.2.5.

For the PROG, the E2-High (high target levels), and the E2 proficiency testing schemes, the “all methods trimmed mean” (ALTM) still serves as the official target. This target calculation includes results of all participants and is heavily dominated by different immunological methods. For E2-High and PROG, with less than five regular LC-MS participants, only a mean value with no statistics was made available by the scheme providers. Therefore, the quality of the LC-MS mean is sometimes questionable for some parameters, especially for PROG, as there were sometimes only two or three participants with only limited consensus between participants, especially at lower concentrated samples (<1 nmol/L).

For the regular E2 scheme—with usually more than 10 regular LC-MS participants—the group mean should be of better quality and the scheme operator also provided statistics. Generally, the E2 official ALTM target was from 10 to 15% lower on average than the LC-MS group mean, clearly demonstrating that immunological methods are delivering different results. This was also reflected by the average bias of our method against both targets, showing much better accordance with the LC-MS group, with a bias of +2.7% compared to a bias of −9.5% against the ALTM target. The SD of bias against the ALTM target of the normal E2 parameter was twice as high as the SD calculated against the LC-MS target. Since the ALTM target is heavily dominated by immunological results, this indicates that the performance of LC-MS methods is much less affected by single sample characteristics (E2 concentration, matrix constituents) than that of the immunological methods.

Interday accuracy in the QC samples was determined from repeated measurements over five days. Mean bias values at three normal QC levels and three diluted QCs for LLOQ evaluation are listed in Table 1. All mean bias values were within the ±15% goal. Most analytes performed very good and rarely a bias exceeding ±5% was found. QC level III of ALDO and DHEAS were the only samples with a mean bias larger than ±10%. The analysis of QC data of out-of-validation batches such as proficiency testing runs or prevalidation experiments including the same LOTs as in validation experiments revealed a QC level III bias of 0.7% for ALDO and 3.2% for DHEAS. This indicates that real accuracy at higher levels is much better than in the validation runs for both analytes. A problem with the single QC III vial used for the preparation of the aliquots for validation experiments could be a plausible explanation for the found differences in accuracy.

Analytical recovery had to be assessed by correcting for endogenous steroid content as there is no steroid-free authentic human serum matrix available. Recovery was calculated as depicted in Equation (S1) of Supplementary Materials. The mean recovery obtained at three different spike concentrations is shown in Table S8. All results were within the goal of 80–120% recovery. With an SD of <10% for all analytes from quantifying in six different serum matrices, this experiment shows that the recovery is stable across different specimens and not significantly impacted by the matrix effects.

#### 2.2.5. Selectivity and Specificity

For the qualitative assessment of the matrix effects in the form of ion suppression or enhancement, a postcolumn infusion experiment was conducted as first described by Bonfiglio et al. [26]. This experiment was also used in the sample preparation optimization, comparing HybridSPE treated samples against samples processed by protein precipitation only. To measure the effect of HybridSPE on the phospholipid content in the final extract, the most common phospholipids of the class of glycero-phosphatidylcholines were monitored by recording specific transitions tuned with standard solutions of every compound. A list of all monitored phospholipids together with their respective mass transitions is provided in Table S9, which includes phosphatidylcholines with two fatty acid side-chains (PCs) and corresponding phosphatidylcholines with only one fatty acid side-chains (lyso-PCs)—both with different chain lengths of C_14_ to C_20_.

As an example, the postcolumn infusion chromatograms of ANDRO are depicted in Figure S8 in comparison with the chromatograms from monitoring phospholipids. Without phospholipid reduction, general ion suppression was detected in serum samples compared to a mobile phase injection, with very high signal suppression between 4 and 5 min. Most phospholipids eluted at the same time interval. In the serum matrix treated with HybridSPE, no general matrix effect was detected, but with still some ion suppression present in the 4 to 5 min interval. Phospholipids were heavily reduced with only some early eluting lyso-PC left that eluted in the zone of suppression. The total peak area of all monitored phospholipids was reduced by >99% with the use of HybridSPE, which was in good accordance with previous reports [27]. Almost identical results to the neat matrix were found in the artificial QC matrix, which was not surprising since the QC materials are based on charcoal-stripped serum.

This experiment clearly demonstrated that phospholipids can coelute with steroid analytes on a biphenyl stationary phase, leading to almost complete signal extinction at a certain time interval if no phospholipid removal is applied in the sample preparation. Interestingly, this was not reported by Lindner et al. [9], even though they used protein precipitation only in the sample preparation and a similar LC-MS method with the same stationary phase material and mobile phases. This is maybe the reason why they did not include ANDRO or 11-DOC in their panel. Moreover, no phospholipid interactions were reported by Desai et al. [28] using the same biphenyl column for a multisteroid assay, but with an LLE sample preparation, which is also not efficient in reducing phospholipids according to Neville et al. [27].

Experiments for the quantification of the matrix effects and sample preparation extraction recoveries were based on pre- and postspike experiments first published by Matuszewski et al. [29]. As these experiments were described only for sample preparations, including an evaporation step, the spiking schemes had to be adapted for the protein precipitation sample preparation scheme used in this assay. By using halved volumes for the postextraction spiking step, we found an efficient way of dealing with restrictions in sample volume due to the limit of 100 µL of sample when using the HybridSPE cartridges. It also allowed us to use the same spiking solutions for every spiking step. Because of the fixed composition of the commercial IS solution, we could not make a combined spiking solution for IS and analytes and we had to use an additional volume of 20 µL for the pre-extraction spike compared to the general sample preparation. This caused a slightly different end volume and composition of the extraction mixture, but it was the only way to allow for good equilibration of the pre-extraction spiked analytes in the serum matrix, especially compared to the only alternative—incorporating the analytes into the precipitation solvent.

Compared to the original Matuszweksi publication, we also included two additional sample sets, C and E. Set C was used for the calculation of the ER. By spiking the analyte standard solution (ASS) pre- and the IS postextraction, it was possible to calculate the ER with the IS correction only for the instrumental part of the analysis process. Set E was included for the measurement of the base content of analytes in every sample matrix, which was subtracted from the results before calculating the matrix effects and process efficiency. Set E was only spiked with BS to correct only for the volume difference and organic content in comparison to the other sample sets.

Similar modifications to the Matuszewski method were also explored recently by Bienvenu et al. [30], but again for a sample preparation with an evaporation step. Our approach is more compact, as it uses only one set of samples for the base content estimation, it is optimized for the use with diluting sample preparation methods such as protein precipitation, and it is suitable for small or restricted sample volumes.

Results from pre-postextraction spike experiments are depicted in Table S10. For DHEAS, PROG, and E2, no significant matrix effect (ME) was present. For all other analytes, a clear influence of the serum matrix was found, as only 60 to 80% of the signal of Set A was reached, while 21-DF showed an even higher signal suppression, with an ME of 45%. The IS almost perfectly corrected for the found ME, with an IS-normalized ME between 90% and 100% for all analytes. The relative matrix effect was ≤10% for the ME and for the IS-normalized ME, meaning that the ME results are reproducible and that the assay performs equally well in different serum matrices. Higher relative matrix effects found for CL, CN, DHEAS, and TES at the low-level spiked samples were considered as not representative because they highly correlated with the ratio of the spike concentration to the base concentration of the spiked sample and were the only samples that had an average ratio of <1 (see Figure S9).

The ER was good for most analytes. Only 11-DOC, E2 and, ALDO had an IS-normalized ER from 80 to 90%. All other analytes had an IS-normalized ER of >90%. Moreover, the relative ER was very good, with no value exceeding 8%. The samples affected by a bad ratio of spiked concentration to base concentration in the spiked samples showed no such effect in the ER estimation, further proving that the high variation was caused only by the disadvantageous choice of spike levels.

The process efficiency (PE) of the assay was mainly dominated by instrument matrix effects and not so much by sample preparation, as indicated by the results for the ME and ER. For most analytes, from 30% to 50% of the signal is lost in the analytical process compared to a neat solution. However, the IS is affected in the same way as the analyte and corrects for these effects very well. The IS-normalized PE values are within ±15% of the 100% goal. The only exceptions are the low-spike samples of some analytes, as described above.

#### 2.2.6. Interferences

The method was validated for the selectivity against thirteen structurally related isobaric steroids. All substances were tested at high nanomolar or micromolar concentrations for better peak identification. A screened compound was considered an interference if it produced a signal in the assay’s ion transitions around the RT of the respective analytes (QN, QL, or IS), differences in the noise level are observed, or the branching ratio deviated more than ±30% from the calibrator or the QC samples.

Signals were found for all tested steroids in at least one ion transition of an analyte analyzing neat solutions. For each tested compound, the RT, chemical structure, properties, and ion transitions in which a signal was found are summarized in Table S11. Effects on the accuracy were checked by spiking all three levels of the QC samples with a mix of all thirteen compounds at concentrations of 100 ng/mL or 1000 ng/mL. Results of these experiment are summarized in Table S12. Most substances were clearly separated by chromatography or had no influence on the quantification of any analyte and could therefore be excluded as interferents. Compounds that were only partially separated from isobaric analytes or influenced quantitative results are further discussed below.

The 17α-estradiol (17α-E2) was not sufficiently separated from its stereoisomer E2 (17β-estradiol), with a RT difference of only 0.05 min. Quantification would be affected if a significant amount of 17α-E2 was present in a serum sample, as demonstrated in the spiking experiment (Table S12). The 17α-E2 was found as a potential interference in other E2 LC-MS/MS assays before [31,32,33,34]. An interference of 17α-E2 can be easily identified by the peak shape, shifted RTs, and by a different branching ratio (E2: 5; 17α-E2: 15). To our knowledge, there are no reference intervals for serum 17α-E2, as it seems that it is detected only in very low levels (<5 pmol/L) or is undetectable in human serum or other human specimens [35,36,37,38]. Taken together, 17α-E2 is most probably negligible as naturally occurring interference in day-to-day routine analysis of E2, and no case was observed with an interfering peak at the E2 signal in the measurement of the human serum samples during the development and validation of this assay. However, 17α-E2 is used as an active agent in pharmaceuticals [39,40]. Therefore, it seems possible that the samples of patients treated with these medicaments could be affected.

Epitestosterone is a potential isobaric interference of TES and DHEA. It elutes between both analytes and is sufficiently separated at concentrations usually found in humans. However, if epitestosterone was spiked at concentrations far exceeding reference values (3000 nmol/L) to QC samples, the quantification of both analytes DHEA and TES was affected at the lower levels (Table S12). However, no interference from epitestosterone was found in measuring patients’ samples. Epitestosterone can be considered as a theoretical interference that should be monitored carefully but which is most probably negligible.

The IS of 21-DF, 21-DF-d8, coelutes with dexamethasone (DXM) and the quantification of 21-DF was affected by the addition of about 500 nmol/L DXM to the QC samples, leading to falsely low measurements (Table S12). When analyzing the pooled samples from patients undergoing a DXM suppression test (n = 5), 21-DF-d8 had a fronting peak shape which was caused by the DXM, which elutes a little bit earlier. The 21-DF quantification seemed to be not affected in the DXM suppression test samples, as concentrations of up to 20 nmol/L were measured in patients after receiving 1 mg dexamethasone about 8 h prior to sample collection for an overnight dexamethasone suppression test [41,42]. The RT of DXM was confirmed by analyzing a pure solution and by monitoring a specific MRM transition of 393 → 147 while measuring the DXM samples mentioned above. An in-source fragmentation of dexamethasone leading to the Q1 ion [M + H − HF − H_2_O]^+^ with the same *m/z* of 355 as 21-DF-d8, and also the same fragment ion with an *m/z* of 319, was described previously [43]. When recording the Q1 spectra and product ion spectra of DXM, a precursor ion of 355 and a fragment ion of 319 were found, confirming this mechanism leading to the interference in the 21-DF-d8 MRM transition. DXM was not described before as an interference for 21-DF quantification. Since the IS is affected, no QC mechanism, such as monitoring branching ratios, can be applied. However, the interference can be circumvented by using a different IS from another analyte for the calculation of the 21-DF results. If near-eluting CN-d8 or ALDO-d4 was used in the analysis of the spiked QC samples, the accuracy was good with both alternative ISs and well within ±15%. Therefore, results from patients with known DXM treatment should be interpreted carefully and, if necessary, results should be calculated using another IS. Otherwise, falsely low 21-DF results cannot be excluded.

Prednisolone, prednisone, and 18-hydroxycorticosterone are known isobaric or isotopic interferences for CL, CN, and ALDO [44,45]. ALDO was chromatographically separated from all three compounds and was not affected. CL was not fully separated of prednisolone, but even at very high prednisolone concentrations, no interference in quantification of CL was observed (see Table S12). Good separation of prednisone and CN was achieved, and prednisone spiked at high levels had no influence on CN accuracy. The 18-OH-corticosterone eluted between CL and CN and was expected to affect quantification in a similar way as described with epitestosterone. However, as it turned out, 18-hydroxycorticosterone was undetectable in the negative ESI mode used for the quantification for CL and CN.

As shown in Table S11, ANDRO should not be affected by the addition of norandrostenedione. It has a mass difference of 18 u, and it is sufficiently separated with a RT difference of 0.15 min. Nevertheless, a signal was observed at the same RT as ANDRO when analyzing a pure solution of norandrostenedione. When looking at a specific mass transition, however, it was obvious that the commercially available norandrostenedione solution was contaminated with ANDRO. Therefore, high bias found for ANDRO by spiking QC samples can be ignored (Table S12).

#### 2.2.7. Stability and Robustness

The maximum column back pressure was very constant with the used column ranging from 410 to 450 bar. Overall maximum back pressures observed were reached directly after starting the pumps. If performing a “cold start” without preheating the column, the back pressure could exceed the pressure limit of 600 bar proposed by the manufacturer (>700 bar observed). After a cold start, a shift in the RTs were noticed, leading to worse separation for some frequently observed interferent peaks. Therefore, the column was preheated offline in the column oven while simultaneously purging and equilibrating the LC system with the mobile phases for the steroid assay. Furthermore, the column life was extended by using a guard column of the same stationary phase type. If handled with care, the Restek biphenyl columns used in the development of this assay showed excellent RT stability and robustness over more than 2000 injections per column (including blank injections). After about 2500 injections, the columns were changed for a new one. The guard column was changed more frequently, after about 500–800 injections. Since the calibrator and control materials are IVD-CE-certified, the stability was set by the manufacturer to three months at −20 °C after reconstitution and aliquoting. Prepared samples were found to be stable for at least 3 days at room temperature.

### 2.3. Assay Comparison

The new LC-MS/MS method was developed to serve in the future as a substitute for already-established commercial IVD-CE-certified immunoassays (IA) used for routine analysis of steroid congeners. To avoid patient result bias due to possible method bias contributions, it is mandatory in the process of the measurement procedure replacement to perform method-comparison measurements. Hence, the novel LC-MS/MS assay was compared to the established IAs by measuring single and pooled patient samples. In addition, data from the UK NEQAS proficiency testing scheme were used to evaluate the measurement bias found in the patient sample-based platform comparison experiment. All data were compared by Passing and Bablok regression analysis [46] and Bland and Altman plots [47].

**Figure 4:**
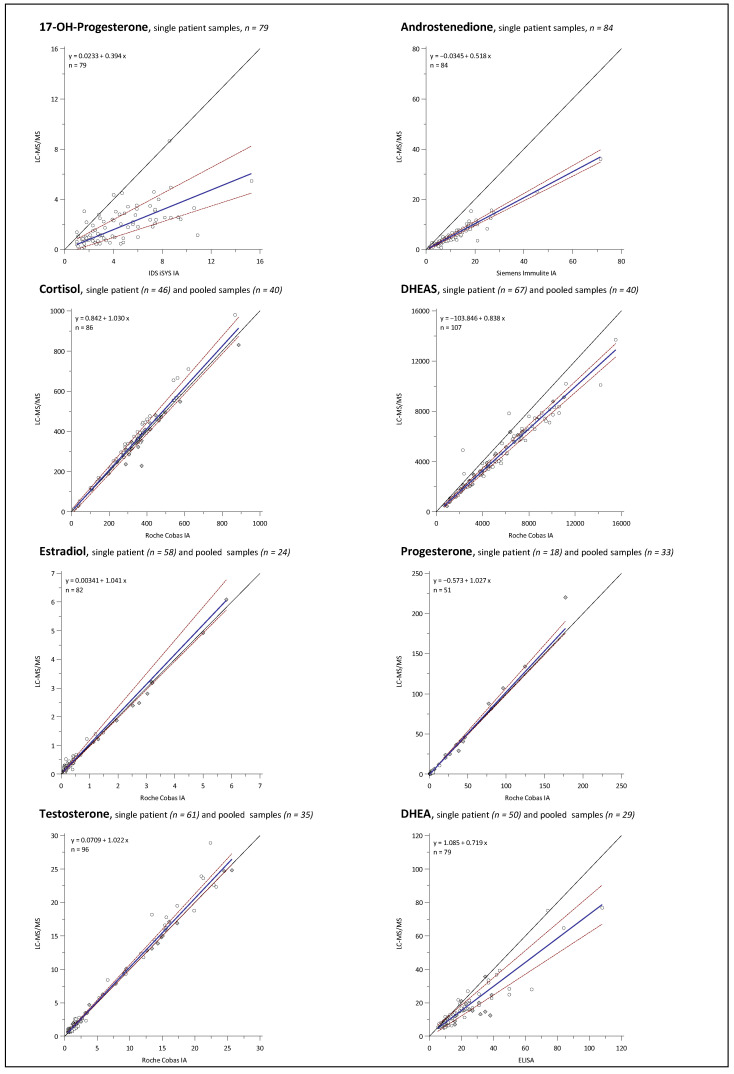
Passing Bablok comparison of the LC-MS/MS method (*y*-axis) and in-house immunoassays (IA, *x*-axis). All results are plotted in nmol/L. Results from patient serum samples are illustrated as circles, from pooled serum samples as squares. Regression line is depicted in blue, 95% CI as dashed lines in red, and line of equality (*x* = *y*) in black.

Comparison plots using Passing and Bablok regression analysis are illustrated in Figure 4 and the results for intercept, slope, and correlation coefficient, together with the respective 95% confidence interval (CI), are summarized in Table 3. In the Passing and Bablok comparison, two methods are considered in good agreement, if the intercept is not different from 0 (CI includes 0) and the slope is not different from 1 (CI includes 1) [48]. An intercept not including 0 indicates a systematic bias, while a slope significantly different from 1 indicates a proportional bias [49]. Good overall method agreement was found for CL, E2, and TES on the Roche IA platform, with neither slope nor intercept statistically different from 1 or 0, respectively. The IAs for the analysis of the DHEA from Demeditec, DHEAS from Roche, the ANDRO from Siemens, and of 17P from IDS all have slope coefficients significantly below 1, indicating a high proportional positive bias to the LC-MS/MS method, as the intercept coefficients are not different from 0 and almost all data points are on the IA’s side of the line of equality. The only assay showing a non-negligible difference from 0 for the intercept coefficient was the PROG assay from Roche (95% CI: −0.68 to −0.46), while the slope was not different from 1 (95% CI: 0.99 to 1.08). This indicates that PROG has some form of systemic bias against the LC-MS/MS assay but still maintains an acceptable mean bias.

**Figure 5:**
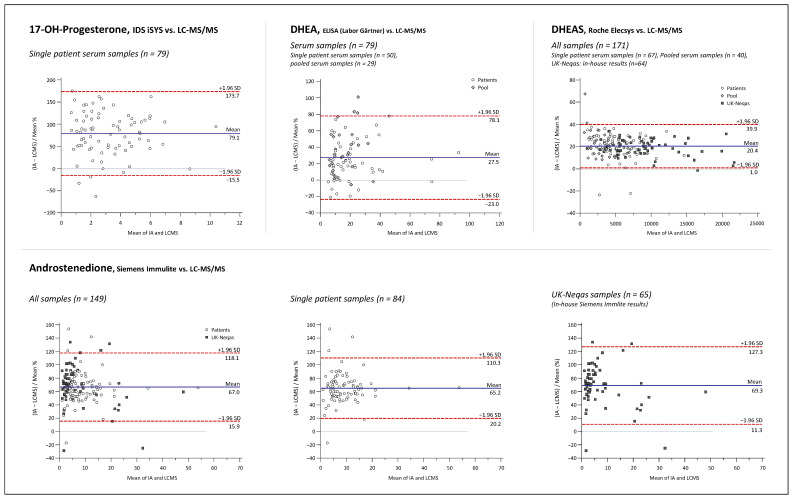
Bland and Altman plots comparing measurement results of the LC-MS/MS (LCMS) method and in-house immunoassays (IA) for analytes 17-OH-progesterone, androstenedione, dehydroepiandrosterone (DHEA), and dehydroepiandrosterone sulfate (DHEAS), differentiating between single patient serum samples (circles), pooled serum samples (diamonds), and UK NEQAS proficiency testing samples (squares). *x*-axis: mean of IA and LC-MS results in nmol/L; *y*-axis: difference between both measurements in % of the mean of both methods.

**Figure 6:**
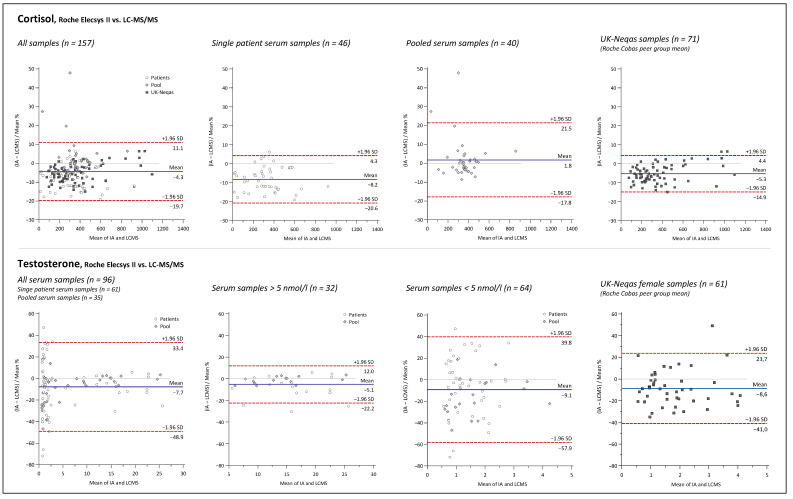
Bland and Altman plots comparing measurement results of the LC-MS/MS (LCMS) method and in-house immunoassays (IA) for analytes cortisol and testosterone differentiating between single patient serum samples (circles), pooled serum samples (diamonds), and UK NEQAS proficiency testing samples (squares). *x*-axis: mean of IA and LC-MS results in nmol/L; *y*-axis: difference between both measurements in % of the mean of both methods.

**Figure 7:**
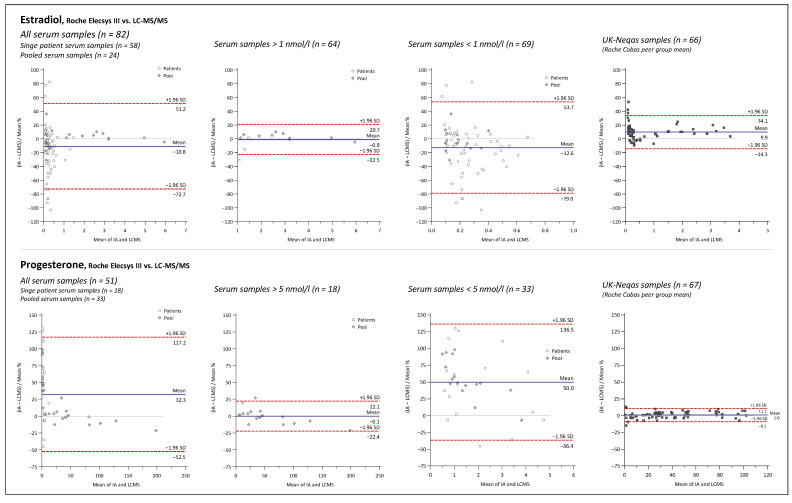
Bland and Altman plots comparing measurement results of the LC-MS/MS (LCMS) method and in-house immunoassays (IA) for analytes estradiol and progesterone differentiating between single patient serum samples (circles), pooled serum samples (diamonds), and UK NEQAS proficiency testing samples (squares). *x*-axis: mean of IA and LC-MS results in nmol/L; *y*-axis: difference between both measurements in % of the mean of both methods.

**Figure 8:**
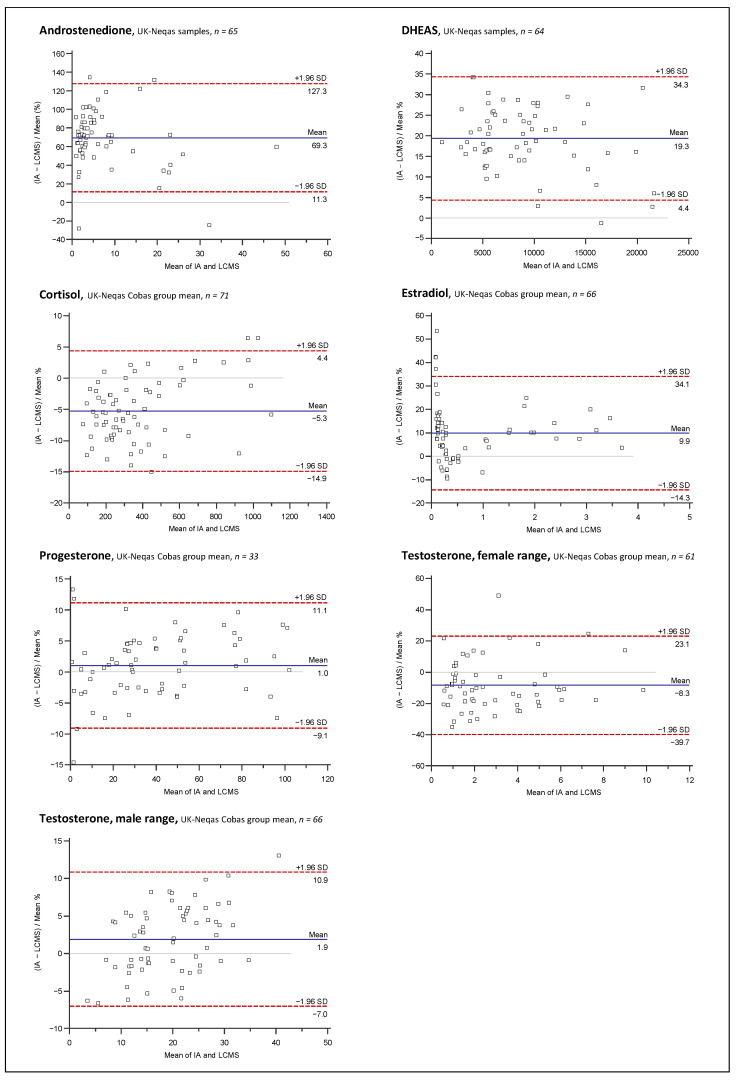
Bland and Altman plots comparing results from measuring proficiency testing samples of the UK NEQAS scheme. Measurement results of the LC-MS/MS (LCMS) method are plotted against results from in-house immunoassays (IAs) (androstendione, Siemens Immulite; dehydroepiandrosterone sulfate (DHEAS), Roche Cobas) or against the mean of all schemes participants using the Roche Cobas assay (all other plots). *x*-axis: mean of IA and LC-MS results in nmol/L; *y*-axis: difference between both measurements in % of the mean of both methods.

Bland and Altman plots comparing the LC-MS/MS method and IAs for 17P, ANDRO, CL, DHEA, DHEAS, E2, PROG, and TES are depicted in Figure 5, Figure 6, Figure 7 and Figure 8.

Overall mean percent differences were at least acceptable (<±15%) only for the Roche Elecsys ECLIA for analytes CL, E2, and TES. For DHEA and DHEAS, the results of the immunological methods were 27.5% and 21.5% higher on average, respectively, demonstrating significant overestimation for both assays compared to the LC-MS/MS. Opposing the results of the 17P IA from IDS and of the ANDRO IA from Siemens with the LC-MS/MS results revealed even higher mean differences of +78.7% and +66.0%, respectively. If looking at the sample subgroups, E2 > 1 nmol/L, PROG > 5 nmol/L, and TES > 5 nmol/L were in quite good agreement with the LC-MS/MS, with mean differences of −0.9%, −0.1%, and −4.5%, respectively. For the results below these thresholds, mean differences were more significant, with −13.5% and −10.1% for E2 and TES, respectively, and much worse for PROG with +45%. Furthermore, at lower concentrations, a much higher variation in the differences between the LC-MS/MS and the IA results was notable for these three analytes, as indicated by the much broader agreement intervals (1.96 SD interval) below the chosen concentration thresholds than above. High variations of result differences over the whole concentration range, with agreement intervals of >±50%, were observed in the comparisons of 17P, ANDRO, and DHEA IAs against the LC-MS/MS.

Interestingly, the method agreement for CL was different between the IA and LC-MS/MS when measuring pooled serum and single-patient samples, with mean differences of +1.8% and −9.4%, respectively. No such matrix-dependent differences were found among the method comparisons for the other analytes.

Furthermore, to evaluate how representative our findings are, data from UK NEQAS proficiency testing scheme were used for Bland and Altman comparisons of the LC-MS/MS method to the Roche Elecsys assays for DHEAS, TES, CL, PROG, and TES, and to the Siemens Immulite assay for ANDRO. Independent measurement results for UK NEQAS samples by in-house IAs were available for DHEAS and ANDRO, whereas for the other analytes, the results of the LC-MS/MS measurements were compared with the Roche Cobas peer group mean according to the UK NEQAS reports. Mean differences and variation of differences were almost identical for DHEAS and ANDRO when measuring the UK NEQAS samples and patient serum samples. For CL, PROG, and TES (male and female), similar results were also obtained in both datasets. Interestingly, E2 results of the Roche Cobas peer group had an overall average difference of +9.9%, while an overall negative mean difference of −11.3% was found in the comparison against the in-house Roche IA with patient serum samples. This was caused mainly by opposing trends of rising bias at lower concentrations.

## 3. Discussion

### 3.1. Method Development

In the present work, it was demonstrated that the newly developed multianalyte method is suitable to measure the steroids 11-DF, 11-DOC, 17P, 21-DF, ANDRO, CC, CL, CN, DHEA, DHEAS, PROG, E2, and TES in human serum in a routine clinical setting. During method development, the occurrence of the ion yield drops due to coeluting lipids proved to be particularly challenging. Interestingly, this phenomenon was not described for technically similar steroid assays using a biphenyl column published earlier, even stating no influence by phospholipids [9,28]. The use of a phospholipid-specific SPE in the sample work-up protocol has resulted in a reduction of the matrix background, so that the residual matrix effects could be compensated by the coeluting the stable-isotope-labeled ISs. Previous reports on assays for quantifying steroids from biological matrices using the HybridSPE technology in the sample preparation have shared mixed findings for extraction recovery or in general performance compared to other procedures such as supported liquid extraction (SLE) or SPE [50,51,52]. In these studies, exact sample preparation procedures either resemble those proposed by the manufacturer or HybridSPE is integrated in a multistep sample-preparation scheme. However, our new approach, using a single-step procedure combining methanolic ZnSO_4_ precipitation with HybridSPE filtration, led to extraction recovery rates of >80% for all steroids and ISs included in our assay. With this sample preparation procedure, a trained person can prepare at least 72 samples in 2 h and, if using a 96-well format, it would also be easy to automate with a potentially even higher sample throughput. Therefore, this method is excellent for the analysis of steroid congeners in a routine clinical setting.

The use of patient samples to evaluate chromatographic separation performance, which had already occurred during method development, allowed the mobile phase gradient to be designed in such a way that interferences could be separated chromatographically as far as possible and that known isobaric steroids such as 11-DF, 21-DF, and CC could be clearly distinguished. In some cases, in addition to separation at the HPLC level, differences in the ionization behavior of the interfering substances were used to render the analyte peaks free of interference.

A perennial problem in steroid analysis is the sensitivity of the analytical setup. In addition to the use of NH_4_F as an ionization-enhancing additive to the mobile phase, the technique of signal addition (transition summing) was used to lower the detection limit for critically low concentrated E2. Based on serial dilution experiments, LLOQs were set to 0.1 nmol/L for all analytes except E2 (15 pmol/L), CN (1 nmol/L), DHEA (5 nmol/L), CL (15 nmol/L), and DHEAS (140 nmol/L), covering biological reference ranges.

### 3.2. Assay Validation

Interassay imprecision was found to be below 5% for all analytes except CN, ALDO, DHT, and E2, ranging between 5% and 10%. For 21-DF, average CV values were around 10% for all QC levels. Similar results were found in human serum samples; hence, a matrix contribution to the measurement uncertainty was not found. Measuring patient samples, the assay delivered reproducible results over five days, reaching CVs below 15% for each analyte, except for two 11-DOC samples with near-LLOQ concentrations measured with a precision from 15% to 20%. The measurement of the available CRMs for TES, E2, CL, and PROG yielded excellent results for the TES, with a mean bias of −0.1% and 2.4% to the target values, and revealed a significant bias for CL and PROG, which was not found in the QC materials and was independent from the utilized calibrator lot. Hence, it seems possible that the value assignment of the calibrator and QC material of the manufacturer might be the cause of this issue. Taking part in the proficiency testing scheme of UK NEQAS for steroids over multiple distributions (n = 24) revealed a good to very good accordance with other LC-MS methods. Average bias was below ±5% for all parameters except PROG, which revealed a more significant positive bias of +9.8% compared to the LC-MS peer group mean, respectively. This bias is in accordance with the bias found in CRM measurements. Regarding longitudinal measurement inaccuracy, only ALDO showed a high scatter, indicating an instable assay performance.

The validation has proven that, in the developed test setup, ALDO and DHT were at the analytical performance limit. Therefore, these analytes were not recommended for routine use in this setting, leaving 11-DF, 11-DOC, 17P, 21-DF, ANDRO, CC, CL, CN, DHEA, DHEAS, PROG, E2, and TES as the recommended steroid analyte panel. For ALDO, coelution with E2 has proven to be a hindrance, as much of the available MS/MS recording time was consumed by using transition summing for E2. Consequently, the ALDO sMRM transitions do not have enough ion collection time, which negatively affects the sensitivity and stability of the ALDO quantification. For DHT, it is the analyte itself that causes problems. The potential formation of a hemiketal and/or geminal diol under the given solvent conditions, and the rather poor ionizability of DHT, make quantitative analysis in the biological reference range rather impossible [6,53]; at best, semiquantitative statements can be made. For DHT, therefore, an analytical strategy is clearly recommended that is accompanied by a concentration of the analyte, e.g., by LLE or SLE.

Ion suppression in patient samples was qualitatively assessed via postcolumn infusion experiments. The effect reduction upon phospholipid removal via offline SPE was clearly visible in the experimental readouts. A spiking experiment based on protocols by Matusewski et al. [29] led to the identification of affected matrices (only patient samples) and to the identification of affected analytes. Recovery calculations did, however, prove that the reduction of the IS signal yield balanced the analyte signal loss very well.

Interference testing resulted in additional peaks in the LC-MS/MS analyses. Trueness of analyte quantification was, however, not affected. A hitherto unreported interference from DXM in 21-DF quantification due to coelution with the IS 21-DF-d8 is presented in this study. It is recommended to test for DXM interference in any LC-MS assay covering 21-DF or its isobars CC and 11-DF if a d8 IS is used. Furthermore, recording CL and CN in the negative mode proved to be effective in prohibiting interference from prednisolone, prednisone, and 18-OH-corticosteorne, despite missing chromatographic separation. Since this phenomenon was reported by others as well [7], and sensitivity was sufficient to cover reference ranges, we recommend using negative ionization for the quantification of CN and CL in the future.

### 3.3. Assay Comparison

Evaluating the performance of established routine in-house IAs by comparison to the newly developed LC-MS/MS method revealed acceptable agreement and performance over the whole concentration range only for CL measured by the Roche Elecsys IA and, at higher serum levels, also for E2 (>1 nmol/L), PROG, and TES (both >5 nmol/L).

The different bias trend in the pooled samples (positive) compared to the single-patient samples (negative) for CL could not be fully rationalized. One reason could be day-to-day method performance differences of one or both methods, since the pooled samples were measured on a single day by both methods, while single-patient samples were measured over a longer period of more than one year. No specific characteristics of the pooled samples could be identified that correlated with proportionally higher measurements by the IA. Influence by gender, pregnancy, serum protein levels, or renal health status on the bias of CL IAs against the LC-MS/MS was observed in previous studies, especially for the Roche assay [54,55].

Although TES and E2 still maintained an acceptable mean bias of <±15%, also at lower concentrations, the increasing variability of result differences indicates lower sensitivity and rising influence of lacking selectivity and specificity due to cross-reactivity, making these assays quite unreliable for the measurement of TES in females, of E2 in men or postmenopausal women, or in children for both analytes. All these patient groups are covered in better analytical quality by the new LC-MS/MS method, especially considering its low bias against CL, E2, and TES measuring higher order reference materials, also demonstrating its superior analytical performance for analytes with a rather good IA performance.

The Roche IA for PROG performed even worse at lower concentrations, making it rather useless for measurements in patients with expected serum levels below 5 nmol/L (e.g., men). It seems to be mainly a specificity issue, but also calibration could be affected, since Passing Bablok regression indicated a systemic bias. It must be noted that the LC-MS/MS methods revealed a bias of about 10% of its own against reference materials for PROG. Therefore, the bias against the true value of the PROG IA could be even higher than as it presented itself against the LC-MS/MS method.

DHEAS, which is also measured on the Roche platform, had a significant positive bias, which was quite constant throughout the tested concentration range, with a similarly narrow interval of difference variation as found for CL. This seems to be an issue of missing standardization to reference methods and should have only limited effect on clinical decision-making if adapted reference intervals are used.

In previous reports, and by comparison to data from the UK NEQAS scheme, similar or almost identical results were obtained for CL, DHEAS, PROG, and TES, which further substantiates the validity of the results in the present study [56,57,58]. The DHEA assay revealed suboptimal performance against the LC-MS/MS method. Not only a significant mean positive bias was observed but also a high variation of results. This indicates major specificity issues, probably due to the cross-reactivities of the antibodies used.

Comparing 17P and ANDRO results of the respective IAs of manufacturers IDS and Siemens to the newly developed LC-MS/MS assay revealed unacceptable performance for both assays. A huge overestimation and a very high variability in result differences was found. In the light of high accuracy of the LC-MS/MS method in the validation experiments (UK NEQAS proficiency testing), it seems obvious that the found differences are mainly caused by analytical problems of the IA. Several previous studies and data of the UK NEQAS scheme are available for the Siemens assay, which all clearly substantiate our findings for the high inaccuracy in measuring ANDRO [56,59,60].

However, to our knowledge, this is the first study comparing the IDS-iSYS 17P IA to a mass spectrometric method. Nevertheless, inaccuracy of 17P results from various IAs were reported before (CLIA, ELISA, RIA). It seems that most IAs used for 17P quantification are quite prone for cross-reactivities. [60,61,62,63,64,65].

For both assays, the clinical usability is somewhat enhanced by using adapted reference intervals, which are also much higher than intervals reported for the LC-MS/MS. Anyway, due to the high variability of the results, these assays do not deliver the needed reliability on which clinical decision making should be based. Especially in cases where diagnosis and treatment are highly dependent on laboratory results, such as in mild cases of hyperandrogenism (PCOS, late-onset CAH), these assays could lead to the wrong diagnosis or therapy decision.

In this study, only single IAs for every analyte were compared with mixed findings regarding the analytical performance of the assays. There are initiatives of IA manufacturers for further enhancements by calibration against reference methods. For example, a new assay for ANDRO was announced by Roche that demonstrated much better agreement with LC-MS/MS than previous IAs [59].

Based on these findings, it is highly recommended to replace IAs that are currently used at our laboratory for ANDRO, DHEA, and 17P quantification as soon as possible. This is probably also reflected by the high share of participants in the UK NEQAS scheme using MS-based methods for 17P and ANDRO parameters. For all other parameters, LC-MS participants are a minority amongst all participants.

## 4. Materials and Methods

### 4.1. Substances and Consumables

NH_4_F and ZnSO_4_ were obtained in analytical grade from Merck/Sigma Aldrich (Buchs, Switzerland). Purified water was produced in-house by a Merck Millipore Direct-Q 3UV water purification system. LC-MS-grade methanol and acetonitrile were from Biosolve BV (Valkenswaard, The Netherlands) and purchased via Chemie Brunschwig (Basel, Switzerland). HybridSPE^®^ phospholipid removal cartridges were purchased from Merck/Sigma Aldrich (Buchs, Switzerland). Pipettes (Multipette Plus; Reference 2, 100–1000 µL; Research, 10–100 µL; Research, 5000 µL) were purchased from Eppendorf (Schönenbuch, Switzerland). Further details on consumables can be found in Table S13.

A set of calibrators consisting of six levels plus a zero-calibrator and a 3-level set of QCs (“MassChrom^®^ Steroids in Serum/Plasma” LC-MS/MS analysis kit), both covering all 15 analytes (11-DOC, 11-DF, 17P, 21-DF, ALDO, ANDRO, CC, CL, CN, DHEA, DHEAS, DHT, E2, PROG, and TES), were purchased from Chromsystems (Munich, Germany). An IS solution containing stable-labeled isotopes of all analytes and lyophilized steroid-free serum were also obtained from Chromsystems. Secondary higher-order reference standards for CL (ERM-DA192, ERM-DA193), E2 (BCR-576, BCR-577, BCR-578), and PROG (ERM DA-347, BCR-348R) were purchased from Merck/Sigma Aldrich, and for TES (ERM-DA345a, ERM-DA346a), from LGC (Wesel, Germany).

Proficiency testing samples for the UK NEQAS scheme were acquired from Birmingham Quality (Birmingham, UK). Additional reference standard materials of analytes named above were obtained from Cerilliant (Merck/Sigma Aldrich) as solutions, which are described in more detail in Table S14. Possible interferents androsterone, dexamethasone, epiandrosteron, epitestosterone, 17α-estradiol, estrone, estriol, etiocholanone, 18-hydroxycorticosterone, norandrostenedione (19-Norandrost-4-ene-3,17-dione), prednisone, prednisolone, testosterone acetate, and trenbolone were purchased as neat substances or in solution from different sources, which are detailed in Table S15.

### 4.2. Preparation of Reagents and Mobile Phases

As precipitating reagent, a 62 mmol/L methanolic ZnSO_4_ solution was prepared freshly on each analysis day by mixing an aqueous 310 mM ZnSO_4_ stock solution (55.6 g/L m/v, stable for 3 months) with pure methanol in a ratio of 1:5, leading to a final methanol concentration of 80% *v*/*v*. Mobile phase A was prepared by mixing 50 mL methanol and 950 mL water and adding 1 mL of a 200 mM NH_4_F stock solution (7.4 g/L m/v, stable for 6 months), and mobile phase B was prepared by adding 1 mL of the NH_4_F stock solution to a fresh 1 L bottle of LC-MS-grade methanol.

### 4.3. Calibrators, Controls, and Serum Samples

Calibrator and QC materials were obtained as lyophilized materials and were reconstituted according to the manufacturer’s instructions. After the addition of 3 mL of water and shaking for at least 20 min on a roller mixer, each material was aliquoted to 150 µL into 1.5 mL reaction tubes and stored at −20 °C until use (stable for 3 months). For the extension of the calibration at the lower end, the QC level I material was diluted 1:5 and 1:20 with steroid-free matrix. The same samples and additional 1:10 and 1:40 dilutions were prepared independently for determining LLOQs.

Human serum samples were gathered from deidentified leftover material from the laboratory’s sample archive. All samples utilized in method development and validation were pools from at least two individual samples to guarantee complete anonymization.

### 4.4. Sample Preparation

Before sample preparation, all samples were brought to room temperature, preferably on a roller mixer or by letting them stand for at least an hour, and brief vortexing (Scientific Industries Vortex-Genie 2, Bohemia, NY, USA) before pipetting.

To 100 µL of calibrator, QC or serum samples 20 µL of IS solution and 200 µL of methanolic ZnSO_4_ solution were added. After vigorous mixing (10 min, 2000 rpm, 15 °C) on a thermoshaker (Eppendorf Thermomixer C), precipitated proteins were separated by centrifugation (10 min, 14,000× *g*, 4 °C) with a Hettich Mikro 400R (Hettich AG, Bäch, Switzerland). The clear supernatant (280 to 300 µL) was pipetted into HybridSPE cartridges for phospholipid removal and pressed through the filter layer by applying centrifugal force, 3 min at 100× *g* followed by 1 min at 500× *g* with a Hettich Rotanta 460RS centrifuge (Hettich AG). The eluate (200 to 240 µL) was transferred to HPLC vials with a microinsert and 40 µL were injected into the LC-MS/MS system for analysis.

### 4.5. Liquid Chromatography–Mass Spectrometry

LC-MS/MS analysis was performed on an Agilent 1290 Infinity II chromatographic system (binary pump, autosampler, and column oven) in conjunction with a Sciex 6500+ triple quadrupole mass spectrometer equipped with an Ion-Drive Turbo-V source. A biphenyl analytical column (Restek Raptor Biphenyl, 2.7 µm, 2.1 × 100 mm, BGB Analytik, Boeckten, Switzerland) with a guard column of the same material (Restek Raptor Biphenyl EXP guard column, 2.7 µm, 2.1 × 5 mm, BGB Analytik) was used for chromatographic separation and was kept at a temperature of 50 °C in the column oven. Mobile phase A was based on 5% methanol and mobile phase B on pure methanol. NH_4_F was used as an additive at a concentration of 0.2 mM in both mobile phases. Gradient elution was programmed to start at 47.5% mobile phase B, linearly increasing to 94% B for 5.3 min, followed by a washing step at 100% B for 0.5 min and ending with re-equilibration to starting conditions at 47.5% for 1.2 min. The autoinject procedure containing a needle wash and sample aspiration (injection volume 40 µL) took about 1 min, adding up to a total run time (injection to injection time) of 8 min. The LC-stream was guided to the ion source only between 0.8 min and 5.3 min, and otherwise—by switching a six-port valve—directly to the waste to reduce contamination of the MS instrument.

### 4.6. Optimizing Mass Spectrometer Settings

Mass spectrometry was performed in the selected reaction monitoring (SRM) mode. Ion transitions for quantification (QN transition) and for qualitative confirmation (QL transition) were investigated and optimized by infusing pure solutions of the analytes into the LC-stream kept at 50% mobile phase B via a T-connector with a syringe pump (“post column infusion”). Tuning mix solutions 1 and 2 (Chromsystems), including all analytes and ISs of the target steroid panel, were used for this experiment. For isobaric analytes (17P, 11-DOC, and CC; 21-DF and 11-DF; DHEA and TES; CN and ALDO), single solutions were also infused for confirmation of the results obtained by using the tuning mix. The most highly abundant ion transitions were tuned to their optimal values for collision energy (CE), declustering potential (DP), entrance potential (EP), and collision cell exit potential (CXP). As a last step, ion source parameters were optimized to the chromatographic flow conditions stated above. All data can be found in Tables S1 and S2. Ion trace recording was performed in the sMRM mode and details on the settings are summarized in Table S3.

### 4.7. Data Recording and Processing

LC-MS raw data recording and processing was performed using Sciex Analyst software. Version 1.6.3 on Microsoft Windows 7 and Version 1.7 on Windows 10 were used. Intelliquant MS3 algorithm was used for integration of peaks. A 3-number smooth was applied to all transitions. Noise level was set to 100% and peak splitting factor to 0. If automatic integration did not recognize the peak or the baseline of the peak correctly, manual integration was used. A minimum peak height was defined for every mass transition. Calibration functions for quantification were obtained by regression analysis over six to eight calibration points using a linear or quadratic model and 1/x^2^ weighting, depending on the analyte. Details on the final calibration functions are summarized in Table S4.

For standard statistical analysis of results, Microsoft Excel (data aggregation, calculation of means, and SDs) was used in different versions (2013, 2016, 365) on Windows and Mac operating systems. Medcalc version 20 (Medcalc Software Ltd., Ostend, Belgium) running on Windows 10 was used for performing advanced statistical analysis, and the generation of plots and graphics (Bland Altmann, Passing Bablok, boxplot) and R-scripts were executed in RStudio version 1.1.1463 (RStudio Inc., Boston, MA, USA) using R programming language version 4.0.3 (The R Foundation for Statistical Computing, Vienna, Austria) on macOS High Sierra 10.13.6.

### 4.8. Method Validation

#### 4.8.1. Linearity and Calibration Model

To identify the best-fit calibration model and evaluate linearity over the whole calibration range, linear and quadratic regression models with different weighting factors (1/x, 1/x^2^) were investigated by assessing residual plots and bias sums. Therefore, a dilution series was prepared in triplicate, each measured independently under individual calibrations. The series was created by mixing the highest and lowest calibrator in a seven-step scheme (calibrator one to calibrator six ratio—1:8, 2:6, 3:5, 4:4, 5:3, 6:2, and 8:1), leading to nine samples total, including calibrators one and six. These sample sets were measured independently under individual calibrations.

#### 4.8.2. Sensitivity

The sensitivity of the assay was defined as a lower limit of quantification (LLOQ) in a multistep process. First, a technical LLOQ was determined experimentally based purely on statistical criteria. Then, targets for a working LLOQ also considering additional fitness for purpose criteria were predefined and finally evaluated in the main multiday validation experiment (see Section 2.2.2.).

The technical LLOQs of the assay were defined as the lowest concentration where an imprecision expressed as a coefficient of variation (CV) of ≤20% and a bias of ≤±20% was reached. CV and bias were calculated from independent measurements (three days, three preparations, triplicate injection) of a sample set consisting of QC level I to III, and QC level I diluted 1:3, 1:5, 1:10, 1:20, 1:40, and 1:100 with steroid-free matrix using the peak area ratio of the analyte and IS. Fitness for purpose criteria considered for defining working LLOQs were biological reference ranges, medical decision points, calibration range, and method performance in authentic serum samples.

#### 4.8.3. Precision and Accuracy

Repeated measurements of a set of samples were performed on five days. A sample set consisted of eight calibrators; three aliquots of three levels of commercial QCs (Chromsystems) plus three aliquots of dilutions of the lowest QC level (1:5, 1:10, 1:20); 10 different human-age- and gender-stratified serum pools; human serum reference materials that were available for TES (ERM-DA345a and ERM-DA346a), PROG (ERM-DA347, BCR-348R), E2 (BCR-576, BCR-577, BCR-578), and CL (ERM-DA192, ERM-DA193). For every sample, the intra- and interday precision was assessed as the coefficient of variation (CV, %), calculated from the mean of all according measurements and the SDs.

#### 4.8.4. Proficiency Testing

For external QC and further accuracy evaluation, we participated in the UK NEQAS scheme for steroids (Birmingham Quality, Birmingham, UK) [66,67]. Results from distributions 470 (November 2019) to 493 (January 2022) were used for this study. Each distribution consisted of three to five individual samples per analyte (30 to 43 sampler per distribution); samples were provided for analytes CL, 17P, PROG, E2 (normal and high panel), TES (female and male panel), ANDRO, DHEAS, and ALDO. Usually, samples were of male- or female-human-serum origin, either pooled or of a single donor. Series of spiked or gradually mixed samples for recovery or linearity experiments were included as well as samples spiked with different concentrations of known interferences. For every sample, only the value of the assigned parameter was reported to UK NEQAS and a bias was calculated against the target value, which was usually the mean of all participants using LC-MS/MS as their method. For parameters PROG, E2, and High-E2, the official target was the “all methods trimmed mean” (ALTM) calculated from all reported values including immunological methods, but the mean value of the LC-MS/MS group was provided as well.

#### 4.8.5. Recovery

Six human serum pools were spiked with recovery spiking solutions (RSSs) at three different concentration levels (low, medium, high) or a blank solution (50% methanol). RSSs were prepared by diluting stock solutions in a multistep process according to Scheme S2 to give the target spiking concentrations depicted in Table S16. All diluting steps were carried out using 50% methanol as diluent. The lowest level was chosen either for a reasonable ratio between the native and the added concentration or for that which should have been at least 10× the LLOQ [68]. Final recovery samples were prepared from 950 µL of serum or steroid-free matrix spiked with 50 µL of RSS or blank solution. After inverting all samples several times and putting them on the rolling mixer for two hours for good mixing, they were put in the fridge overnight at 4 °C for further equilibration. On the next day, samples were portioned to give 6 aliquots with 150 µL each and were then stored in the freezer (−20 °C) until analysis.

#### 4.8.6. Selectivity and Specificity—Postcolumn Infusion Experiments

In general, the postcolumn infusion experiments were conducted as first reported by Bonfiglio et al. [26]. A standard solution in methanol containing all analytes (“Tuning Mix Solutions 1 & 2”, Chromsystems) was infused with a syringe pump (10 µL/s) directly into the LC effluent stream via a T-connector. This generates a constant signal at the detector for every MRM transition monitored without changing the RT or other parameters of the measurement procedure significantly. After the injection of a sample, the analyte signal is altered during a run by matrix constituents eluted from the column. A signal enhancement or suppression around the RT of the target analyte compared to signal intensities before sample injection indicates an influence by matrix effects. Samples injected during this experiment consisted of mobile phase only (blank), steroid-free serum (calibrator matrix), or pooled patient serum (male and female serum pools) and were worked up with and without the phospholipid reduction step (HybridSPE). Depending on the analyte, concentrations of the standard solution were adapted to give signal intensities which are usually found within the calibration ranges. Tuning mix solutions 1 and 2 were mixed 1:1 and further diluted with methanol. Analytes DHEA, DHEAS, DHT, CL, and CN were measured with a final dilution factor of 1:20, and for all other analytes, a 1:200 dilution was used. In addition to analytes and ISs, specific mass transitions of common phospholipids were tuned and subsequently monitored during each infusion experiment.

#### 4.8.7. Matrix Effects and Extraction Recovery

Experiments for the evaluation and quantification of the matrix effect (ME), extraction recovery (ER), and the overall process efficiency (PE) were based on the publications by Matuszewski et al. [29]. The term ME is used in this context for signal suppression or enhancement caused by parts of the serum matrix during chromatography, ionization, or mass spectrometry in comparison to a neat matrix. The ER parameter describes the efficiency of the sample preparation step. With the third parameter, PE, the complete measurement procedure is evaluated by comparing the signal response of the serum samples to a neat sample with the same amount of analyte added. These parameters are calculated such that the value should be near 100% if the measurement is not affected by matrix effects or poor extraction recovery.

Since the experiments needed to calculate these parameters were described only for sample preparations including an evaporation step, the spiking schemes had to be adapted for the sample preparation used in this assay. The limited sample volume of 100 µL due to the use of the HybridSPE cartridges was also a factor that had to be considered.

Six pooled human serum samples and steroid-free matrix were spiked either before (“pre-spike”) or after (“post-spike”) sample preparation with the IS solution (ISS), with a blank solution (BS) (water/methanol 1:1) and/or with two levels of an analyte standard solution (ASS) containing all 15 analytes (Table S16). The prespike of the ASS and the BS was added the day before analysis for better equilibration in the serum matrix as described in the recovery experiment. Prespike of ISS was added during sample work up as it would be in the general sample preparation. Due to volume losses in the sample preparation, the postspike was always performed with half the volumes used for the prespike, which allowed to use the same ASS and ISS for both spiking steps. Five sets of samples (A to E) had to be prepared. For each set, a different spiking scheme was used, which is depicted in Table S17. The final ME, ER, and PE parameters—both absolute and IS-corrected—were calculated as the mean from the individual values of each sample. All calculations were based on the formulas using the peak area of the analyte and/or the IS of selected sample sets (A to E) described above (Equations (S1)–(S7)).

#### 4.8.8. Interferences

During method development, validation, and comparison, all measured samples were screened for possible interferences by optical evaluation of chromatograms (additional peaks or altered peak shape in comparison to QC samples). Including UK NEQAS samples, more than a thousand serum samples were screened.

Thirteen available steroids, listed in Table S15, were investigated as possible interferents, either because they have identical molecular masses or were reported previously as interferents. Pure solutions of each steroid were worked up and injected at high concentrations (10, 100, and/or 1000 ng/mL), and all ion transitions were recorded in classical MRM mode covering the whole run-time to identify the RT of every tested compound. To check the potential influence on accuracy, the same steroids were also spiked to QC level I to III to give a final concentration of 10 or 100 ng/mL.

#### 4.8.9. Stability

Stability of the worked-up samples at room temperature was studied during the interday experiment. Five sets of samples were measured at five different days over a time span of 10 days and were stored at room temperature afterwards. By analyzing every sample set a second time, stability could be estimated at one, three, six, eight, and ten days after sample preparation by comparing the results with that of the first measurements. Samples were considered as stable if the calculated concentration differed by less than 10% of the initial measurement.

### 4.9. Assay Comparison

#### 4.9.1. Immunoassays

In our laboratory, steroids are currently routinely measured only by commercial immunological methods. Analytes CORT, DHEAS, E2, PROG, and TES are currently routinely measured by electrochemiluminescence (ECLIA) IA “Elecsys” on the Cobas modular platform from Roche Diagnostics GmbH (Mannheim, Germany). ANDRO is measured by solid-phase, competitive chemiluminescent enzyme IA Immulite 2000 from Siemens Healthineers AG (Erlangen, Germany), and 17P by chemiluminescence IA IDS-iSYS from Immunodiagnostic Systems Ltd. (Bolton, UK); DHEA samples are routinely forwarded to external laboratory MVZ Labor Ravensburg (Ravensburg, Germany), where they are measured by an ELISA from Demeditec (Kiel, Germany). Details such as working principle and main performance data of all IAs are summarized in Table S18.

#### 4.9.2. Single-Patient Samples

Single-patient samples were collected from the laboratory archive using serum residuals of routine analysis (usually gel separated serum). Samples were only collected if they already had a value assigned by routine analysis at least for one analyte also covered by the new LC-MS/MS method. Samples were deidentified and stored at −20 °C before remeasurement with LC-MS/MS, which happened continuously as part of sample batches of method development and validation analytical runs over a period of multiple months and using different LOTs of calibration materials and analytical columns (March 2020 to December 2021).

#### 4.9.3. Pooled Serum Samples

Forty samples from pooled human serum only were specially created for this experiment from patient sample leftovers with the target to cover all biological reference ranges as well as pathological values of every analyte, which is usually not possible by random collection of single-patient samples. For this goal, we created two types of working serum pools. The first type of working pools was created from specific, and in some cases rather rare, patient samples with expected concentrations at each end of the reference range of certain analytes, e.g., samples from patients after ACTH stimulation (high CL) or after metapyrone treatment (high 11-DF and CC). The second type of working pools was made from easy-to-collect patient groups such as pregnant women (high E2 and PROG) or children (low E2 and TES). After determining the steroid concentration of all working pools, final serum pools were created by targeted mixing of both types of working pools.

#### 4.9.4. Data Analysis

Results of LC-MS/MS and IA methods were compared by using Bland and Altman plots and Passing Bablok statistical analysis. Only results inside the limits of quantification of either method were considered.

## Figures and Tables

**Table 1:** Average intraday (IM-ALL) and total interday (IM-IN) imprecision and average interday bias (BI-ALL) calculated for every QC level of 11-deoxycorticosterone (11-DOC), 11-deoxycortisol (11-DF), 17-OH-progesterone (17P), 21-deoxycortisol (21-DF), androstenedione (ANDRO), aldosterone (ALDO), corticosterone (CC), cortisol (CL), cortisone (CN), dehydroepiandrosterone (DHEA), dehydroepiandrosterone sulfate (DHEAS), dihydrotestosterone (DHT), estradiol (E2), progesterone (PROG), and testosterone (TES). QC-I 1:20, QC-I 1:10, and QC-I 1:5 are dilutions of QC-I and were included for confirmation of pre-estimated LLOQs. Number of samples: 9 times 5 for intraday imprecision, 45 for interday imprecision, and bias per QC level. Dilution levels below the predefined LLOQ were not analyzed (n.a.).

Analyte	Parameter	Unit	QC-I 1:20	QC-I 1:10	QC-I 1:5	QC-I	QC-II	QC-III
11-DOC		(nmol/L)	0.012	0.023	0.047	0.233	0.590	3.050
	IM-IN	(%)	n.a.	13.5	9.0	4.7	3.3	2.7
	IM-ALL	(%)	n.a.	6.3	5.7	1.9	1.2	0.9
	BI-ALL	(%)	n.a.	14.5	6.9	1.4	−1.8	−1.3
17P		(nmol/L)	0.04	0.09	0.17	0.85	4.30	25.80
	IM-IN	(%)	14.3	7.3	5.3	3.4	2.1	1.2
	IM-ALL	(%)	7.9	6.1	3.9	1.4	0.8	0.6
	BI-ALL	(%)	1.9	0.3	0.6	−0.5	−0.3	−1.6
ANDRO		(nmol/L)	0.05	0.09	0.18	0.91	3.75	31.90
	IM-IN	(%)	11.2	6.0	5.5	2.6	2.0	2.5
	IM-ALL	(%)	6.1	4.5	3.6	1.2	0.8	0.8
	BI-ALL	(%)	5.1	−0.8	1.5	−0.4	1.4	−2.4
DHEA		(nmol/L)	0.3	0.7	1.4	6.8	42.6	144.0
	IM-IN	(%)	n.a.	n.a.	23.9	7.2	5.0	3.4
	IM-ALL	(%)	n.a.	n.a.	11.5	4.3	1.9	2.0
	BI-ALL	(%)	n.a.	n.a.	11.2	−1.2	−0.9	−2.1
DHT		(nmol/L)	0.017	0.033	0.066	0.33	1.590	3.890
	IM-IN	(%)	n.a.	45.5	26.2	8.3	5.8	6.0
	IM-ALL	(%)	n.a.	14.2	10.0	6.3	2.8	3.5
	BI-ALL	(%)	n.a.	91.3	25.4	−1.1	1.4	−2.3
PROG		(nmol/L)	0.05	0.09	0.19	0.94	9.72	47.90
	IM-IN	(%)	21.7	8.0	6.3	2.8	2.0	1.5
	IM-ALL	(%)	4.5	3.8	1.8	0.8	0.4	0.3
	BI-ALL	(%)	20.6	7.8	2.2	−2.5	1.5	−0.4
TES		(nmol/L)	0.03	0.07	0.14	0.68	5.13	26.50
	IM-IN	(%)	18.6	11.1	5.6	3.9	2.3	2.5
	IM-ALL	(%)	13.8	7.6	4.1	2.0	1.1	1.4
	BI-ALL	(%)	−0.8	0.1	2.3	0.2	1.9	0.0
21-DF		(nmol/L)	0.01	0.03	0.06	0.28	1.05	6.58
	IM-IN	(%)	n.a.	n.a.	18.4	10.6	9.6	10.9
	IM-ALL	(%)	n.a.	n.a.	11.3	5.3	3.9	2.1
	BI-ALL	(%)	n.a.	n.a.	6.6	0.3	7.3	5.2
11-DF		(nmol/L)	0.04	0.08	0.17	0.82	4.05	26.5
	IM-IN	(%)	12.3	7.9	4.5	11.0	3.1	2.6
	IM-ALL	(%)	5.7	4.8	3.0	1.8	1.2	0.9
	BI-ALL	(%)	26.1	7.5	0.9	−3.1	−6.9	2.9
CC		(nmol/L)	0.12	0.25	0.49	2.47	12.00	80.40
	IM-IN	(%)	15.6	9.6	5.1	3.5	4.4	3.5
	IM-ALL	(%)	7.6	5.0	3.1	1.2	0.8	1.4
	BI-ALL	(%)	−3.7	1.7	3.4	2.4	5.9	−0.3
DHEAS		(nmol/L)	35	70	140	699	4230	13800
	IM-IN	(%)	5.2	3.4	2.0	2.2	2.1	1.1
	IM-ALL	(%)	0.6	0.6	0.6	0.5	0.4	0.3
	BI-ALL	(%)	38.6	14.9	3.8	−6.6	−5.7	−12.3
E2		(nmol/L)	0.015	0.030	0.059	0.297	1.510	9.260
	IM-IN	(%)	10.2	9.9	9.2	6.1	5.3	4.7
	IM-ALL	(%)	8.8	7.0	7.9	3.7	2.6	2.7
	BI-ALL	(%)	2.4	−2.1	−0.8	−0.5	−1.3	−2.7
CL		(nmol/L)	3	7	14	69	166	487
	IM-IN	(%)	22.7	10.7	4.0	2.6	3.2	2.2
	IM-ALL	(%)	2.5	1.7	1.2	1.0	1.5	1.4
	BI-ALL	(%)	−0.9	−0.6	1.3	0.5	1.6	0.7
CN		(nmol/L)	0.29	0.57	1.15	5.74	34.2	80.8
	IM-IN	(%)	19.4	13.4	9.0	7.1	6.6	6.8
	IM-ALL	(%)	6.3	7.7	5.1	3.6	4.8	4.8
	BI-ALL	(%)	34.5	13.4	4.8	−0.3	1.0	−1.4
ALDO		(nmol/L)	0.013	0.026	0.051	0.255	0.699	2.430
	IM-IN	(%)	n.a.	n.a.	25.4	4.2	7.2	5.5
	IM-ALL	(%)	n.a.	n.a.	9.1	3.7	3.2	2.9
	BI-ALL	(%)	n.a.	n.a.	17.3	6.2	2.9	14.1

**Table 2:** Precision in pooled human serum samples of 11-deoxycorticosterone (11-DOC), 11-deoxycortisol (11-DF), 17-OH-progesterone (17P), 21-deoxycortisol (21-DF), androstenedione (ANDRO), aldosterone (ALDO), corticosterone (CC), cortisol (CL), cortisone (CN), dehydroepiandrosterone (DHEA), dehydroepiandrosterone sulfate (DHEAS), dihydrotestosterone (DHT), estradiol (E2), progesterone (PROG), and testosterone (TES). Serum pools are age- and gender-stratified. F and M are female and male samples, respectively. 1–5 are the age groups <10 years, 10–16 years, 16–45 years, 45–60 years, and >60 years, respectively.

Analyte	Unit	F1	F2	F3	F4	F5	M1	M2	M3	M4	M5
11-DOC	(nmol/L)	0.25	0.20	0.16	0.10	0.10	0.24	0.16	0.14	0.09	0.06
	(%)	5.4	10.8	7.4	13.5	18.9	13.4	5.7	9.0	12.0	9.2
17P	(nmol/L)	2.24	2.15	2.42	1.10	0.87	1.29	1.46	2.61	2.40	2.34
	(%)	1.8	6.2	4.6	4.5	13.0	8.7	2.5	3.6	1.3	1.2
ANDRO	(nmol/L)	0.35	4.54	4.03	2.18	1.83	0.25	1.66	3.08	2.56	1.84
	(%)	2.6	5.9	4.5	3.0	12.9	11.2	1.8	2.6	0.8	1.0
DHEA	(nmol/L)	3.68	17.59	17.89	9.87	6.21	2.68	9.52	14.58	8.09	4.51
	(%)	45.0	7.3	7.4	5.5	12.6	22.1	6.3	7.4	11.5	23.9
DHT	(nmol/L)	n.a.	0.231	0.283	0.138	0.120	0.140	0.569	1.245	0.960	0.940
	(%)	n.a.	23.9	12.3	16.9	16.4	24.7	7.4	7.7	6.6	10.9
PROG	(nmol/L)	46.12	3.98	23.57	1.70	0.13	28.89	0.14	0.25	0.20	0.19
	(%)	0.6	5.8	4.9	3.9	7.5	9.0	5.9	4.4	5.8	4.3
TES	(nmol/L)	0.07	0.97	1.06	0.68	0.76	0.49	7.60	18.49	16.01	14.18
	(%)	10.5	7.9	5.9	5.8	13.5	11.1	1.6	3.1	2.1	2.2
21-DF	(nmol/L)	0.03	0.06	0.06	0.07	0.08	0.02	0.05	0.04	0.07	0.08
	(%)	38.5	19.9	40.4	18.9	22.6	35.1	47.6	28.0	35.4	32.3
11-DF	(nmol/L)	1.23	1.52	0.72	0.81	0.96	0.78	1.48	0.87	1.02	0.85
	(%)	3.4	6.9	5.4	2.5	11.6	10.4	4.9	4.4	2.4	3.4
CC	(nmol/L)	5.13	15.18	11.93	16.27	10.65	6.65	7.91	15.78	10.29	7.36
	(%)	4.6	6.8	5.7	5.2	12.1	7.1	5.2	9.6	4.6	4.3
DHEAS	(nmol/L)	451	4283	4307	2013	1407	419	4153	6115	3189	1744
	(%)	1.3	5.1	4.4	3.2	11.7	7.6	1.5	1.9	1.0	0.7
E2	(nmol/L)	0.393	0.391	2.365	0.102	0.027	0.104	0.029	0.093	0.080	0.079
	(%)	4.3	5.1	5.9	7.9	10.9	11.0	12.4	5.3	8.0	5.9
CC	(nmol/L)	187	355	358	401	406	223	247	343	361	313
	(%)	1.8	5.2	5.3	3.5	11.1	10.2	4.0	2.6	1.9	3.2
CL	(nmol/L)	47.3	68.7	51.8	48.3	49.1	54.2	54.3	52.0	51.6	41.1
	(%)	3.9	7.5	6.6	4.6	12.1	10.3	6.5	7.4	6.1	5.7
ALDO	(nmol/L)	0.271	0.302	0.239	0.214	0.228	0.274	0.243	0.176	0.146	0.189
	(%)	7.1	6.6	8.2	8.1	11.0	8.8	5.6	10.3	5.2	5.7

**Table 3:** Results from Passing and Bablok regression analysis comparing the LC-MS/MS method (*y*-axis) and routine IAs (*x*-axis) for 17-OH-progesterone (17P), androstenedione (ANDRO), cortisol (CL), dehydroepiandrosterone (DHEA), dehydroepiandrosterone sulfate (DHEAS), estradiol (E2), progesterone (PROG), and testosterone (TES).

Comparator Assay ^†^	Analyte	Intercept	(95% CI) of Intercept	Slope	(95% CI) of Slope	R^2^ *	(95% CI) of R^2^	Linear ^§^	n
ECLIA	CL	0.84	(−13.51 to 10.00)	1.03	(1.00 to 1.08)	0.966	(0.948 to 0.978)	Yes	86
ECLIA	DHEAS	−103.84	(−206.60 to 11.90)	0.84	(0.81 to 0.86)	0.974	(0.963 to 0.983)	Yes	107
ECLIA	E2	0.00	(−0.02 to 0.02)	1.04	(0.99 to 1.16)	0.891	(0.835 to 0.928)	Yes	82
ECLIA	PROG	−0.57	(−0.68 to −0.46)	1.03	(0.99 to 1.08)	0.937	(0.892 to 0.964)	Yes	51
ECLIA	TES	0.05	(−0.01 to 0.13)	1.03	(1.00 to 1.06)	0.959	(0.939 to 0.972)	Yes	96
ECLIA	17P	0.02	(−0.28 to 0.33)	0.39	(0.31 to 0.52)	0.670	(0.528 to 0.776)	Yes	79
CLIA	ANDRO	−0.03	(−0.29 to 0.22)	0.52	(0.49 to 0.55)	0.936	(0.903 to 0.958)	Yes	84
ELISA	DHEA	1.09	(−0.34 to 2.30)	0.72	(0.62 to 0.82)	0.897	(0.844 to 0.933)	Yes	79

* Spearman rank correlation coefficient, ^§^ Cusum test for linearity, ^†^ ECLIA and CLIA on-site, ELISA contractor laboratory.

## Data Availability

Raw data to the data presented in this study are available on request from the corresponding author. The data are not publicly available due to logistic reasons.

## Supplementary Materials

The following supporting information can be downloaded at: https://www.mdpi.com/article/10.3390/ijms232314691/s1.
