# Supplementary material for: Design and Validation of a Sensitive Multisteroid LC-MS/MS Assay for the Routine Clinical Use: One-Step Sample Preparation with Phospholipid Removal and Comparison to Immunoassays
Source: Int J Mol Sci. 2022 Nov 24;23(23):14691. doi: 10.3390/ijms232314691 (PMC9736865; doi:10.3390/ijms232314691)
Supplement: Supplementary file 1 [file ijms-23-14691-s001.zip › ijms-2049436-supplementary.pdf]

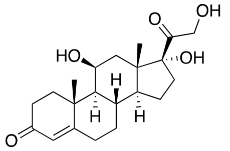

Cortisol (CL)

$C_{21}H_{30}O_5$
M_r = 362.46 g mol^{-1}
CAS Nr 50-23-7

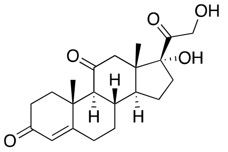

Cortisone (CN)

$C_{21}H_{28}O_5$
M_r = 360.45 g mol^{-1}
CAS Nr 53-06-5

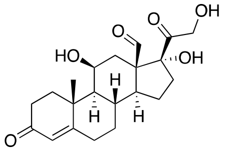

Aldosterone (ALDO)

$C_{21}H_{28}O_5$
M_r = 360.45 g mol^{-1}
CAS Nr 52-39-1

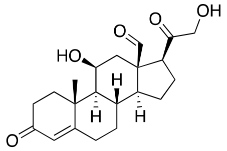

Corticosterone (CC)

$C_{21}H_{30}O_4$
M_r = 346.47 g mol^{-1}
CAS Nr 50-22-6

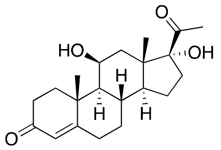

21-Deoxycortisol (21-DF)

$C_{21}H_{30}O_4$
M_r = 346.47 g mol^{-1}
CAS Nr 641-77-0

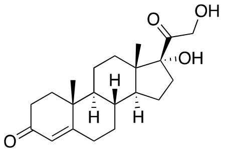

11-Deoxycortisol (11-DF)

$C_{21}H_{30}O_4$
M_r = 346.47 g mol^{-1}
CAS Nr 152-58-9

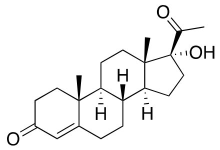

17-Hydroxyprogesterone (17P)

$C_{21}H_{30}O_3$
M_r = 330.46 g mol^{-1}
CAS Nr 68-96-2

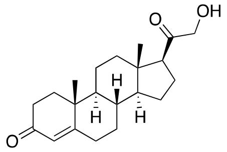

11-Deoxycorticosterone (11-DOC)

$C_{21}H_{30}O_3$
M_r = 330.46 g mol^{-1}
CAS Nr 64-85-7

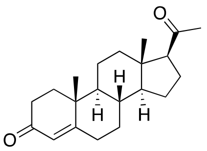

Progesterone (PROG)

$C_{21}H_{30}O_2$
M_r = 314.47 g mol^{-1}
CAS Nr 57-83-0

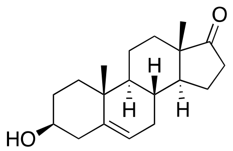

Dehydroepiandrosterone (DHEA)

$C_{19}H_{28}O_2$
M_r = 288.43 g mol^{-1}
CAS Nr 53-43-0

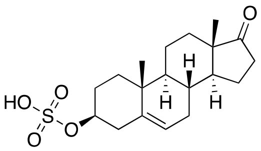

Dehydroepiandrosterone sulfate (DHEAS)

$C_{19}H_{28}O_5S$
M_r = 368.49 g mol^{-1}
CAS Nr 651-48-9

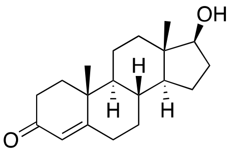

Testosterone (TES)

$C_{19}H_{28}O_2$
M_r = 288.43 g mol^{-1}
CAS Nr 58-22-0

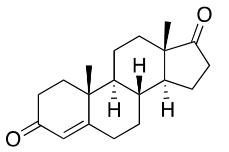

Androstenedione (ANDRO)

$C_{19}H_{26}O_2$
M_r = 286.42 g mol^{-1}
CAS Nr 63-05-8

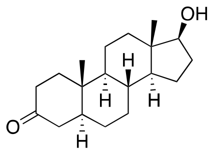

Dihydrotestosterone (DHT)

$C_{19}H_{30}O_2$
M_r = 290.45 g mol^{-1}
CAS Nr 521-18-6

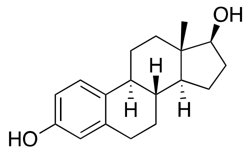

17β-Estradiol (E2)

$C_{18}H_{24}O_2$
M_r = 272.38 g mol^{-1}
CAS Nr 50-28-2

Scheme S1. Steroid analytes covered by the LC-MS/MS assay presented in this work. Chemical structures, sum formula, molecular weight and CAS number is depicted together with the steroid name and the abbreviation used in parenthesis.

Formula used to calculate recovery rates in different experiments are as follows:

$$\text{Recovery (\%)} = \frac{\text{c(total)} - \text{c(unspiked)}}{\text{c(spike)}} \times 100 \qquad \text{(S1)}$$

$$\text{Matrix effect (ME) \%} = \frac{\text{B} - \text{E}}{\text{A}} \times 100\ \% \qquad \text{(S2)}$$

$$\text{Extraction recovery (ER) \%} = \frac{\text{C}}{\text{B}} \times 100\ \% \qquad \text{(S3)}$$

$$\text{Process efficiency (PE)\%} = \frac{D - E}{A} \times 100\ \% \quad \text{(S4)}$$

$$ME_{(\text{IS corrected})} = \frac{ME_{(\text{analyte})}}{ME_{(\text{IS})}} \times 100\ \% \quad \text{(S5)}$$

$$ER_{(\text{post IS corrected})} = \frac{C_{\text{analyte}}\, B_{\text{IS}}}{B_{\text{analyte}}\, C_{(\text{IS})}} \times 100\ \% \quad \text{(S6)}$$

$$PE_{(\text{IS corrected})} = \frac{PE_{(\text{analyte})}}{PE_{(\text{IS})}} \times 100\ \% \quad \text{(S7)}$$

	Stock solutions (D0)		Dilution 1 (D1)		Dilution 2 (D2)	
	M [g/mol]	c [ng/ml]	Dilution factor	c [ng/ml]	Dilution factor	c [ng/ml]
11-DOC	330.46	100000	100	1000	25.0	40
11-DF	346.46	1000000	100	10000	50.0	200
17P	330.46	1000000	100	10000	33.3	300
21-DF	346.46	1000000	1000	1000	25.0	40
ALDO	360.44	100000	100	1000	25.0	40
ANDRO	286.41	1000000	100	10000	33.3	300
CC	346.46	1000000	100	10000	6.3	1600
DHEA	288.42	1000000	100	10000	2.5	4000
DHT	290.44	1000000	1000	1000	156.3	40
E2	272.38	1000000	1000	1000	625.0	10
PROG	314.46	1000000	100	10000	62.5	1000
TES	288.42	1000000	100	10000	625.0	100
CL	362.47	1000000			66.7	15000
CN	360.44	100000			100.0	1000
DHEAS	391.48	1000000				

RSS High			RSS Medium			RSS low		
Dilution factor	c [ng/ml]	c [nmol/l]	Dilution factor	c [ng/ml]	c [nmol/l]	Dilution factor	c [ng/ml]	c [nmol/l]
2.5	16	48.4	10.0	4	12.1	20.0	2.0	6.1
	80	230.9		20	57.7		10.0	28.9
	120	363.1		30	90.8		15.0	45.4
	16	46.2		4	11.5		2.0	5.8
	16	44.4		4	11.1		2.0	5.5
	120	419.0		30	104.7		15.0	52.4
	640	1847.3		160	461.8		80.0	230.9
	1600	5547.5		400	1386.9		200.0	693.4
	16	55.1		4	13.8		2.0	6.9
	4	14.7		1	3.7		0.5	1.8
	400	1272.0		100	318.0		50.0	159.0
	40	138.7		10	34.7		5.0	17.3
2.5	6000	16553.1	5.0	3000	8276.5	10.0	1500	4138.3
	400	1109.8		200	554.9		100	277.4
8.3	120000	306529.1	33.3	30000	76632.3	66.7	15000	38316.1

Scheme S2. Pipetting scheme for the creation of three levels of spiking solutions (RSS) containing all 15 analytes used for the recovery experiment. Stock solutions and dilution 1 (D1) were prepared individually for every analyte. For Dilution 2 (D2) two mixed solutions were prepared, one for multiple analytes from D1 solutions, and one for CN and CL from their stock solutions. RSS high, medium, and low were finally prepared by mixing together both D2 solutions and DHEAS stock solution at different dilution factors. 50% methanol was used as diluent in all steps.

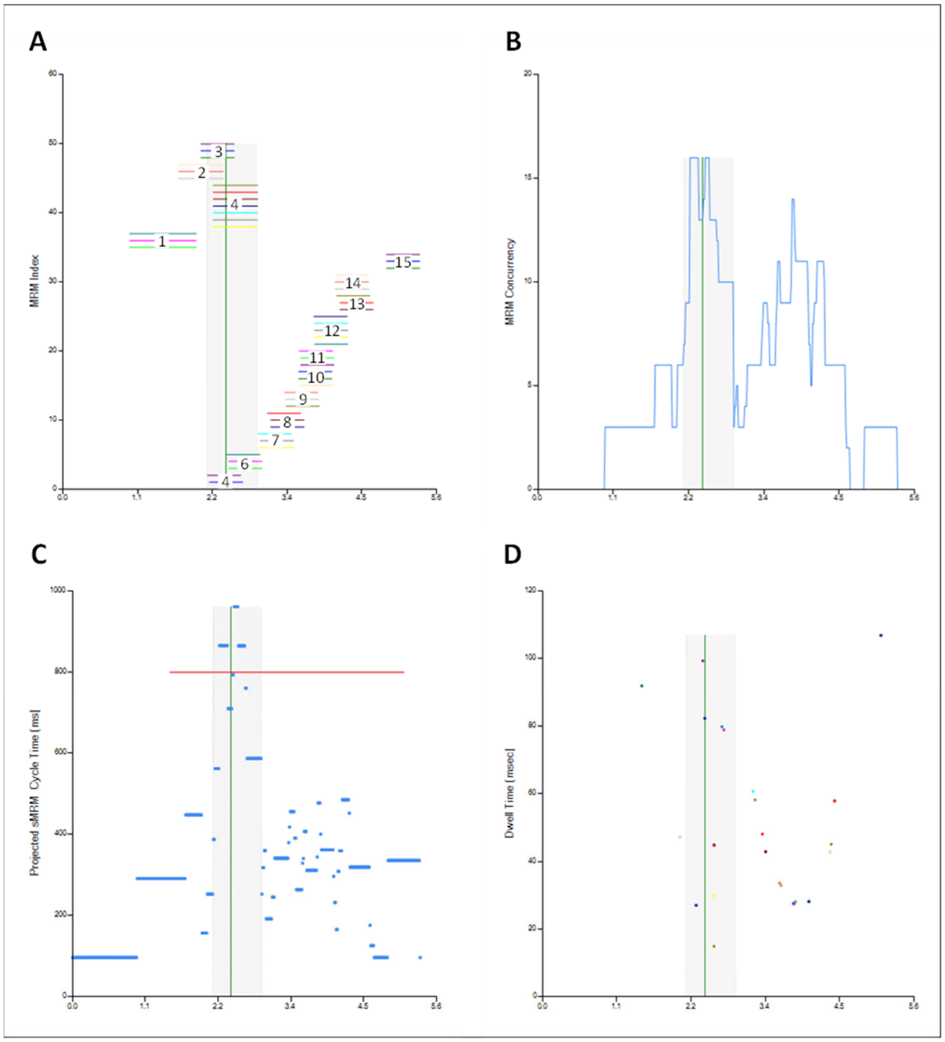

Figure S1. sMRM calculator tool output for 11-deoxycorticosterone (11-DOC), 11-deoxycortisol (11-DF), 17-OH-progesterone (17P), 21-deoxycortisol (21-DF), androstenedione (ANDRO), aldosterone (ALDO), corticosterone (CC), cortisol (CL), cortisone (CN), dehydroepiandrosterone (DHEA), dehydroepiandrosterone sulfate (DHEAS), dihydrotestosterone (DHT), estradiol (E2), progesterone (PROG), and testosterone (TES). (**A**) Time windows of all programmed ion transitions. Analyte numbering: 1 DHEAS; 2 CL; 3 CN; 4 21-DF; 5, 6 E2 and ALDO; 7 11-DF; 8 CC; 9 DHEA; 10.11 TES and 17P; 12 DHT; 13, 14 ANRDO and 11-DOC; 15 PROG; (**B**) number of concurrent MRM transitions; estimated cycle time (**C**) and estimated dwell times (**D**) over one run.

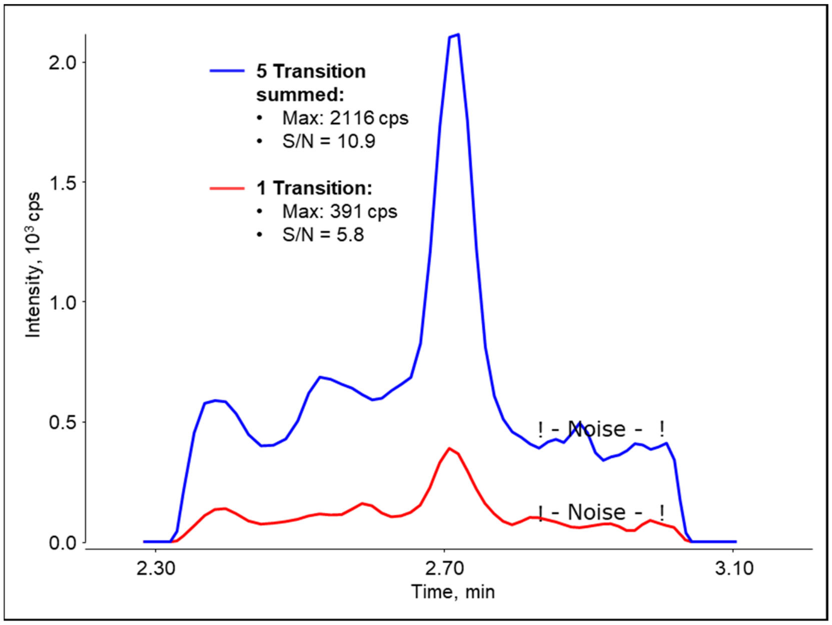

Figure S2. Effect of transition summing. A single ion transition chromatogram (red) overlayed with a chromatogram consisting of 5 summed transitions (blue) of the same sample (QC I 1:10, 30 pmol/L).

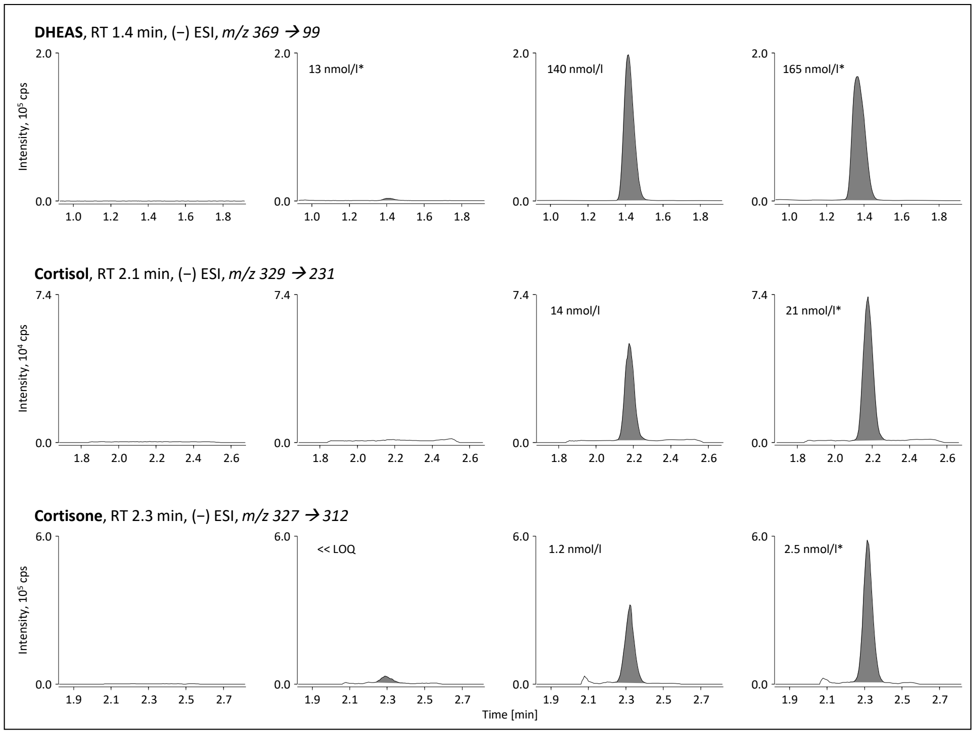

Figure S3. Exemplary chromatograms of the quantifier ion trace of DHEAS, cortisol, and cortisone. Analytes are arranged according to their retention time. Chromatograms show from left to right, a double blank sample (mobile phase) recorded after the highest calibrator, a blank serum sample (calibration matrix), the lowest used calibrator sample and an authentic patient serum sample with a near LLOQ concentration are depicted. Target concentrations are depicted where available, measured concentrations are marked with an asterisk.

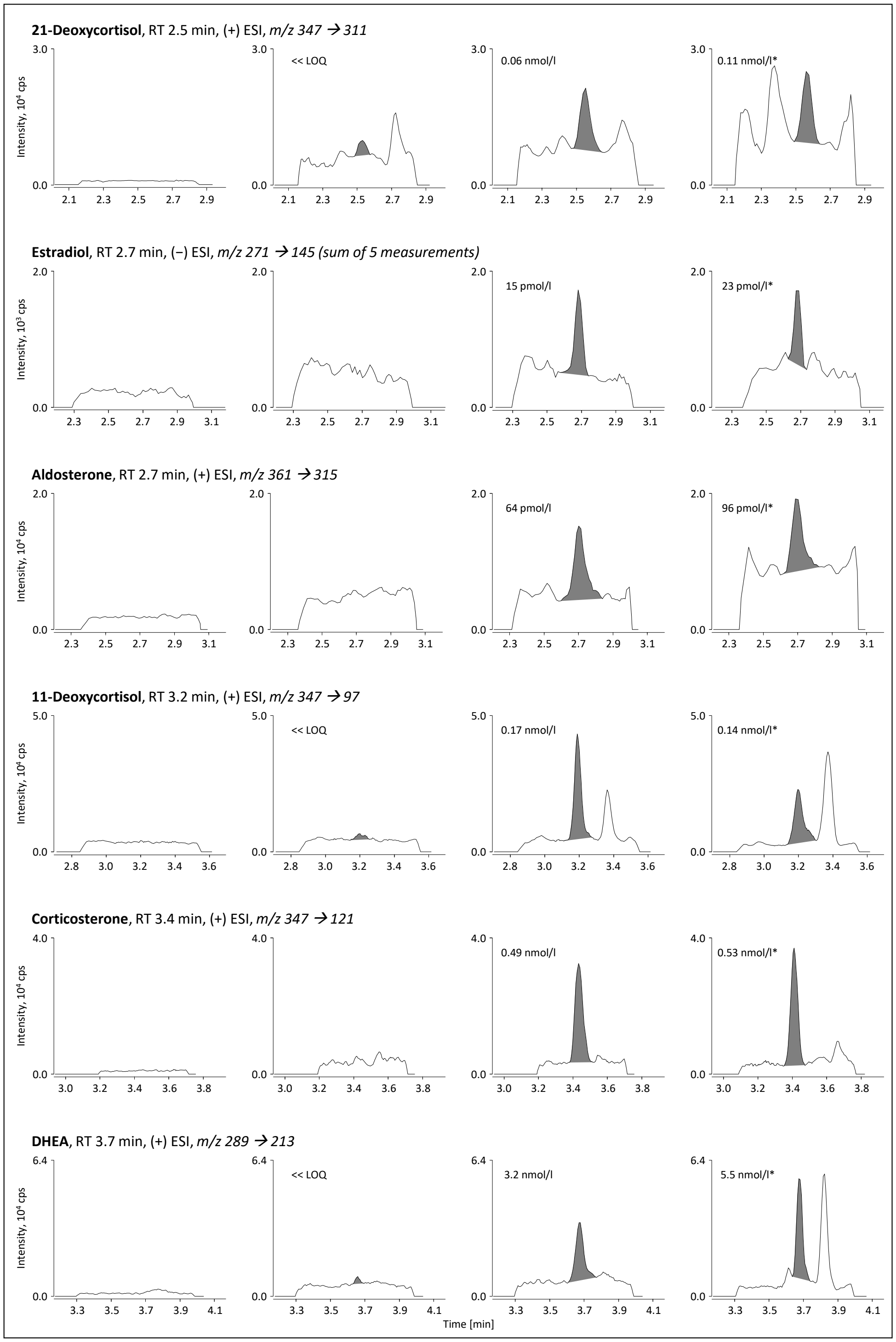

Figure S4. Exemplary chromatograms of the quantifier ion trace of 21-deoxycortisol, estradiol, aldosterone, 11-deoxycortisol, corticosterone, and DHEA. Analytes are arranged according to their retention time. Chromatograms show from left to right, a double blank sample (mobile phase) recorded after the highest calibrator, a blank serum sample (calibration matrix), the lowest used calibrator sample and an authentic patient serum sample with a near LLOQ concentration are depicted. Target concentrations are depicted where available, measured concentrations are marked with an asterisk.

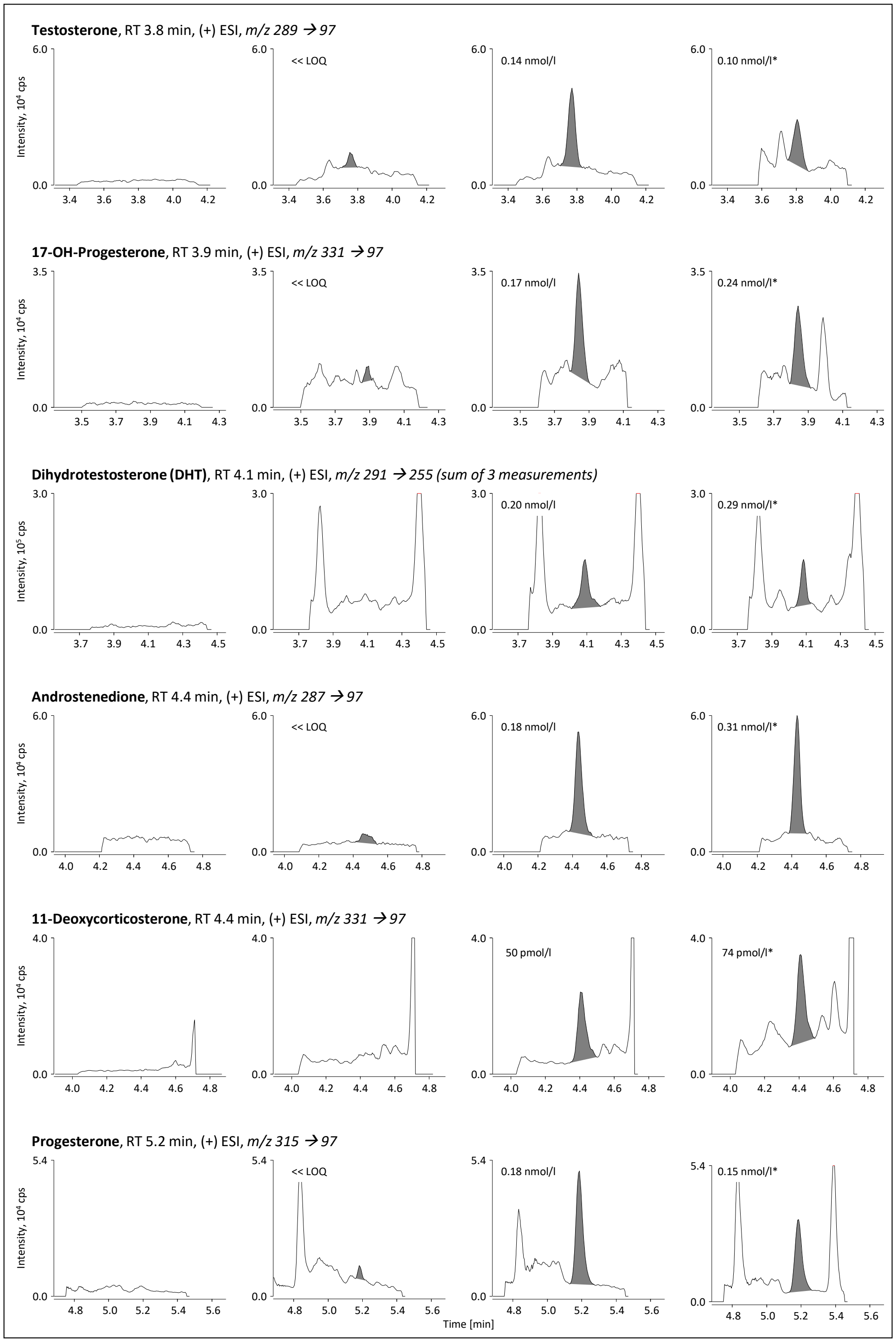

Figure S5. Exemplary chromatograms of the quantifier ion trace of testosterone, 17-hydroxyprogesterone, DHT, androstenedione, 11-deoxycortisone, and progesterone. Analytes are arranged according to their retention time. Chromatograms show from left to right, a double blank sample (mobile phase) recorded after the highest calibrator, a blank serum sample (calibration matrix), the lowest used calibrator sample and an authentic patient serum sample with a near LLOQ concentration are depicted. Target concentrations are depicted where available, measured concentrations are marked with an asterisk.

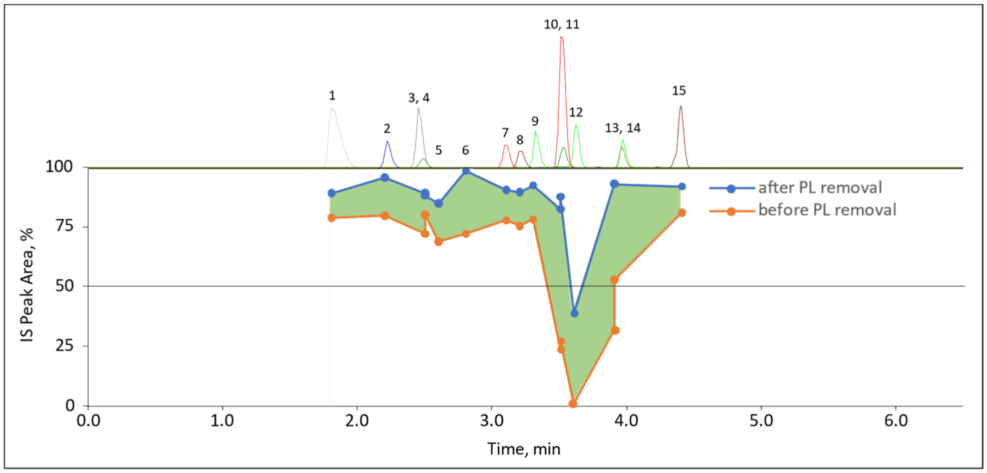

Figure S6. Retention-time dependent IS peak area recovery differences in serum samples before and after removal of phospholipids (PL) for the standards of 11-deoxycorticosterone (11-DOC), 11-deoxycortisol (11-DF), 17-OH-progesterone (17P), 21-deoxycortisol (21-DF), androstenedione (ANDRO), aldosterone (ALDO), corticosterone (CC), cortisol (CL), cortisone (CN), dehydroepiandrosterone (DHEA), dehydroepiandrosterone sulfate (DHEAS), dihydrotestosterone (DHT), estradiol (E2), progesterone (PROG), and testosterone (TES). Average IS peak area in calibrator samples was used as reference for recovery calculation. These data were generated are from a preliminary method with shorter analysis time. An exemplary chromatogram serves as an analyte identifier for each data point. Peak labelling: 1 DHEAS; 2 CL; 3 CN; 4 21-DF; 5, 6 E2 and ALDO; 7 11-DF; 8 CC; 9 DHEA; 10.11 TES and 17P; 12 DHT; 13, 14 ANRDO and 11-DOC; 15 PROG.

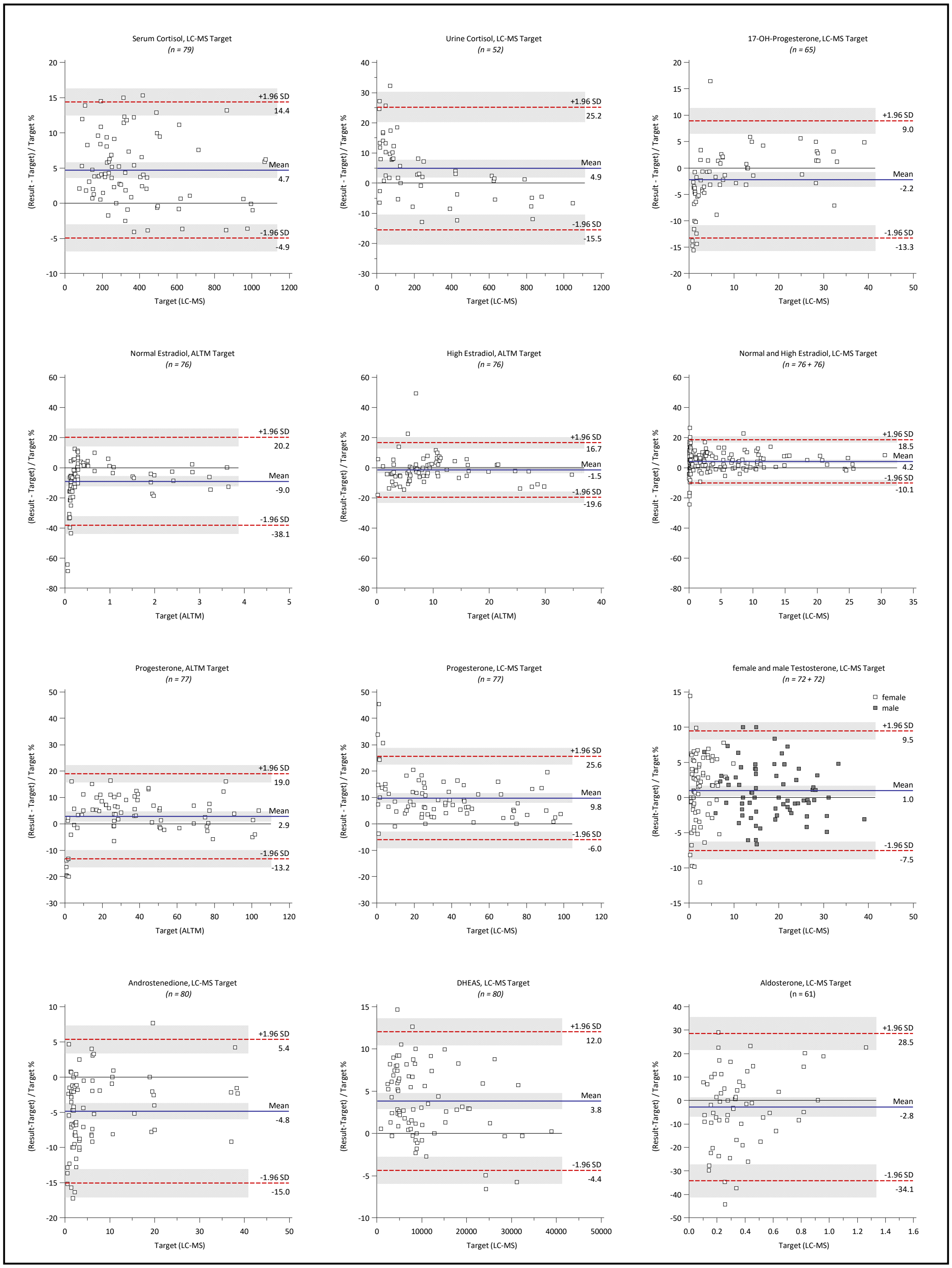

Figure S7. Bland and Altman plots illustrating differences found between the LC-MS/MS method results and target values of the UK-NEQAS proficiency scheme. Data of distributions 470 to 493 included.

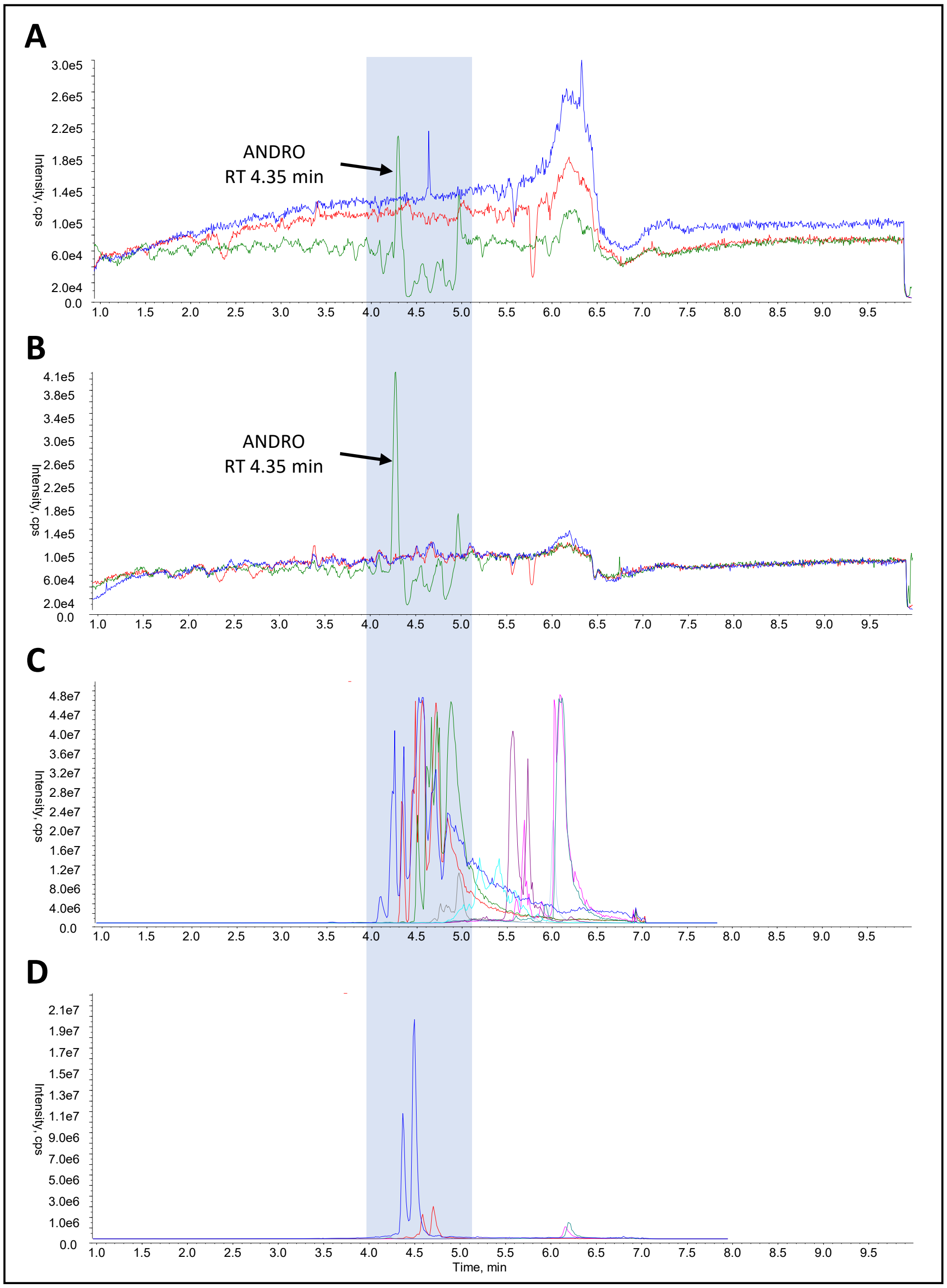

Figure S8. Post column infusion chromatograms of the analyte androstendione (ANDRO) of the same serum sample without (**A**) and with (**B**) the HybridSPE filtration step. Ion trace of serum sample injection is depicted in green, of QC-matrix in red and of mobile phase in blue. In chromatograms (**C**) and (**D**) results of monitoring phospholipid ion traces are shown accordingly. Retention time interval with highest signal suppression is highlighted.

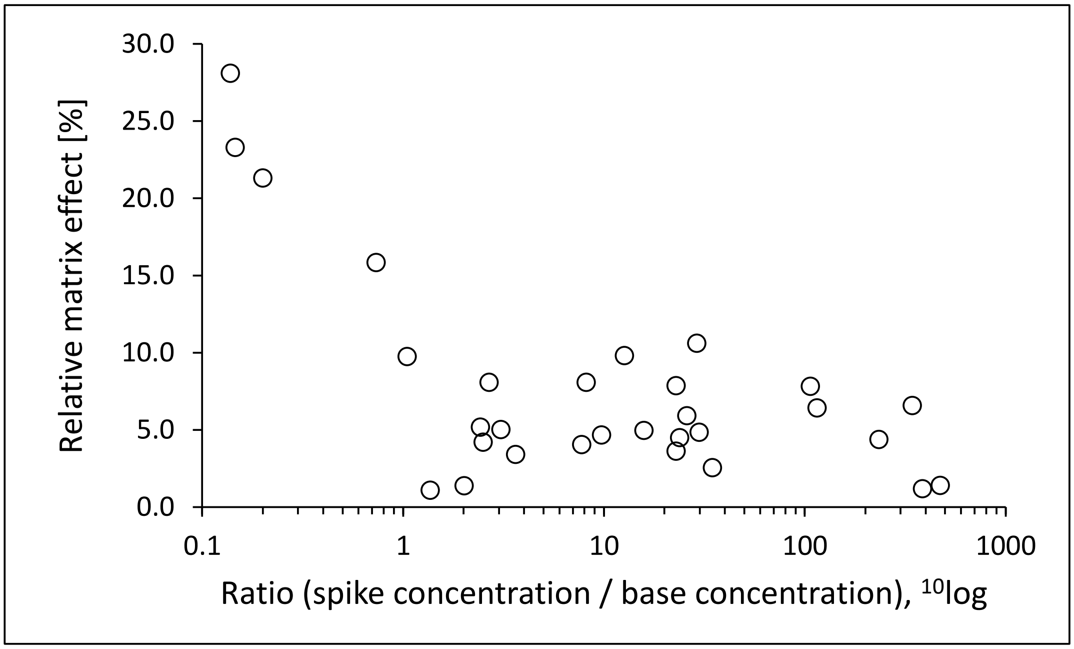

Figure S9. Influence of the spike-to-base concentration ratio on the relative matrix effect.

Table S1. Mass transitions and respective optimized MS-instrument parameters included in the method for the analytes and internal standards of 11-deoxycorticosterone (11-DOC), 11-deoxycortisol (11-DF), 17-OH-progesterone (17P), 21-deoxycortisol (21-DF), androstenedione (ANDRO), aldosterone (ALDO), corticosterone (CC), cortisol (CL), cortisone (CN), dehydroepiandrosterone (DHEA), dehydroepiandrosterone sulfate (DHEAS), dihydrotestosterone (DHT), estradiol (E2), progesterone (PROG), and testosterone (TES)..

					Quantifier			Qualifier		
**Substance**	**RT [min]**	**M [g/mol]**	**Q1-Ion**	**Q1 m/z**	**Q3 m/z**	**DP [V]**	**CE [V]**	**Q3 m/z**	**DP [V]**	**CE [V]**
DHEAS	1.45	368.5	$[M+1]^-$	369.4	99.0	-60	-28	81.9	-60	-105
CL	2.15	362.5	$[M-33]^-$	329.4	231.2	-55	-33	301.2	-55	-29
CN	2.30	360.5	$[M-33]^-$	327.4	312.2	-59	-28	299.1	-59	-28
21-DF	2.50	346.5	$[M+H]^+$	347.3	311.3	65	24	121.1	65	31
E2*	2.68	272.4	$[M-1]^-$	271.3	145.1	-60	-54	183.1	-60	-55
ALDO	2.75	360.5	$[M+H]^+$	361.3	315.2	56	28	343.2	56	24
11-DF	3.18	346.5	$[M+H]^+$	347.3	97.0	65	31	109.1	65	35
CC	3.35	346.5	$[M+H]^+$	347.3	121.1	65	23	109.1	65	24
DHEA	3.65	288.4	$[M+H]^+$	289.3	213.2	70	27	253.3	50	24
TES	3.79	288.4	$[M+H]^+$	289.3	97.1	70	22	109.1	70	24
17P	3.84	330.5	$[M+H]^+$	331.3	97.0	75	28	109.1	75	33
DHT *	4.10	290.5	$[M+H]^+$	291.3	255.3	55	22	159.2	55	31
11-DOC	4.35	330.5	$[M+H]^+$	331.3	97.0	75	28	109.1	75	33
ANDRO	4.35	289.4	$[M+H]^+$	287.3	97.0	100	53	109.1	100	53
PROG	5.10	314.5	$[M+H]^+$	315.3	97.1	40	20	109.1	40	22
DHEAS-d6	1.45	374.3	$[M+1]^-$	375.4	99.9	-60	-30			
CL-d4	2.10	366.5	$[M-33]^-$	333.4	235.2	-55	-33			
CN-d8	2.30	368.5	$[M-33]^-$	332.4	317.2	-59	-28			
21-DF-d8	2.46	354.5	$[M+H]^+$	355.3	319.0	65	24			
E2-d5	2.68	277.4	$[M-1]^-$	276.4	145.0	-120	-73			
ALDO-d4	2.75	364.5	$[M+H]^+$	365.3	319.2	56	28			
11-DF-d5	3.18	351.5	$[M+H]^+$	352.3	100.0	74	30			
CC-d8	3.35	354.5	$[M+H]^+$	355.3	125.2	65	23			
DHEA-d5	3.65	293.5	$[M+H]^+$	294.3	218.3	50	29			
TES-d3	3.79	291.4	$[M+H]^+$	292.3	97.1	73	22			
DHT-d3	4.10	293.5	$[M+H]^+$	294.3	258.3	50	15			
17P-13C3	3.84	333.4	$[M+H]^+$	334.3	100.0	75	28			
ANDRO-13C3	4.35	289.4	$[M+H]^+$	290.3	100.1	100	53			
11-DOC-d8	4.31	338.5	$[M+H]^+$	339.3	100.1	55	28			
PROG-13C3	5.10	317.4	$[M+H]^+$	318.3	100.0	40	20			

* Recorded and summed (transition summing) five times. Programmed by using m/z ratios with a 0.001 u difference for the Q3 quantifier (e.g., 145.098, 145.099, 145.100, 145.101 and 145.102 for E2).

Table S2: Optimized ion source parameters for negative and positive mode ionization

Parameter	Setting
Curtain Gas	35
Collision Gas	10 / medium §
Ion Spray voltage	± 4500 V
Temperature	500°C
Ion Source Gas 1	55
Ion Source Gas 2	55

§ depending on Analyst software version (1.6.3 / 1.7)

Table S3: Scheduled MRM settings and estimated dwell time for the analytes 11-deoxycorticosterone (11-DOC), 11-deoxycortisol (11-DF), 17-OH-progesterone (17P), 21-deoxycortisol (21-DF), androstenedione (ANDRO), aldosterone (ALDO), corticosterone (CC), cortisol (CL), cortisone (CN), dehydroepiandrosterone (DHEA), dehydroepiandrosterone sulfate (DHEAS), dihydrotestosterone (DHT), estradiol (E2), progesterone (PROG), and testosterone (TES).

Analyte	Retention time [min]	Time Window [s]	Target cycle time [s]	Estimated dwell time§ [ms]
Positive ionization				
21-DF	2.45	40		75
ALDO	2.75	40		56
11-DF	3.18	40		40
CC	3.35	40		34
DHEA	3.65	40		25
TES	3.79	40	0.4	21
17P	3.84	40		21
DHT§	4.10	40		22
ANDRO	4.35	40		36
11-DOC	4.35	40		49
PROG	5.10	40		107
Negative ionization				
DHEAS	1.45	60		92
CL	2.10	40		47
CN	2.30	30	0.4	27
E2 quantifier §	2.68	40		45
E2 qualifier	2.68	40		30

§ Dwell times estimated by the sMRM calculator tool provided by Sciex with their Analyst software package.

Table S4: Calibration functions including slope and intercept, used regression model, and weighing factor and mean coefficient of correlation R^2 of independent measurements of a set of calibrators in course of validation experiments (n=5) for 11-deoxycorticosterone (11-DOC), 11-deoxycortisol (11-DF), 17-OH-progesterone (17P), 21-deoxycortisol (21-DF), androstenedione (ANDRO), aldosterone (ALDO), corticosterone (CC), cortisol (CL), cortisone (CN), dehydroepiandrosterone (DHEA), dehydroepiandrosterone sulfate (DHEAS), dihydrotestosterone (DHT), estradiol (E2), progesterone (PROG), and testosterone (TES).

Analyte	calibration range [nmol/L]	Regression	Weight	a (intercept)	b (slope)	c	R^2
				Mean	Mean	Mean	range
11-DOC	0.05–8.63	lin	$1/x^2$	−0.000423	0.165		0.9993–0.9998
17P	0.18–41.60	quad	$1/x^2$	0.001326	0.075	−0.0000647	0.9993–0.9998
ANDRO	0.2–44.3	lin	$1/x^2$	0.000886	0.061		0.9997–0.9998
DHEA	3.2–200.0	lin	$1/x^2$	0.015840	0.009		0.9971–0.9998
DHT	0.165–5.97	lin	$1/x^2$	0.001129	0.278		0.9990–0.9998
PROG	0.2–78.3	quad	$1/x^2$	−0.000041	0.038	-0.0000360	0.9993–1.0000
TES	0.13–39.90	lin	$1/x^2$	0.000742	0.031		0.9988–0.9996
21-DF	0.06–13.7	lin	$1/x^2$	0.003806	0.117		0.9963–0.9998
11-DF	0.2–35.5	lin	$1/x^2$	−0.000257	0.063		0.9992–0.9999
CC	0.5–143.0	lin	$1/x^2$	0.001408	0.063		0.9985–0.9995
DHEAS	160–15629	quad	$1/x^2$	−0.005670	0.000	0.0000000043	0.9989–0.9993
E2	0.015–19.0	lin	$1/x^2$	0.006110	1.216		0.9991–0.9999
CL	14–782	quad	$1/x^2$	0.001674	0.007	0.000000632	0.9994–1.0000
CN	1.1–111.0	lin	$1/x^2$	0.000236	0.029		0.9958–0.9994
ALDO	0.058–7.10	lin	$1/x^2$	0.001994	0.186		0.9936–0.9995

Table S5: Limit of quantification, concentration and molecules on column and concentration of lowest calibration point for the quantifier and qualifier trace of 11-deoxycorticosterone (11-DOC), 11-deoxycortisol (11-DF), 17-OH-progesterone (17P), 21-deoxycortisol (21-DF), androstenedione (ANDRO), aldosterone (ALDO), corticosterone (CC), cortisol (CL), cortisone (CN), dehydroepiandrosterone (DHEA), dehydroepiandrosterone sulfate (DHEAS), dihydrotestosterone (DHT), estradiol (E2), progesterone (PROG), and testosterone (TES).. Substance amount on column was calculated from measured concentration, dilution in sample preparation, extraction efficiency and injection volume.

Analyte	Quantifier			Qualifier	
	LLOQ [nmol/L]	On column amount * [fmol]	Lowest calibrator [nmol/L]	LLOQ [nmol/L]	Lowest calibrator [nmol/L]
ALDO	0.10	1.1	0.064	0.3	0.064
ANDRO	0.10	1.1	0.18	0.10	0.182
CC	0.50	5.6	0.50	0.50	0.50
CL	15.0	169	13.9	15.0	13.9
CN	1.0	11.2	1.15	1.0	1.15
11-DOC	0.05	0.56	0.05	0.20	0.046
11-DF	0.10	1.1	0.17	0.10	0.17
21-DF	0.10	1.1	0.06	0.20	0.06
DHEA	5.0	56.2	3.43	5.0	3.43
DHEAS	140	1575	140	140	140
DHT	0.30	3.3	0.20	0.50	0.45
E2	0.015	0.17	0.015	0.10	0.06
17P	0.10	1.1	0.17	0.10	0.17
PROG	0.10	1.1	0.19	0.10	0.19
TES	0.10	1.1	0.14	0.10	0.14

* On column amount calculated from LLOQ in fmol/µL × 40µL injection volume / 3.2 for dilution in sample preparation x 90% (average absolute extraction recovery determined in pre/post spike experiment) / 100.

Table S6: Results for bias and precision measuring ERM human serum reference materials with IVD-CE certified calibration materials during assay validation. Mean values of inter-day measurements (n = 5) are compared with the target values (bias) and the inter-day measurement uncertainty (imprecision) was calculated.

**Analyte**	**Parameter**		**Certified Human Serum Reference Material**		
			**Low**	**Medium**	**High**
Testosterone	Sample Identifier:		ERM DA-346a	- -	ERM DA-345a
	certified value ±SD	[nmol/l]	0.89 ± 0.12	- -	19.09 ± 0.56
	found mean ±SD	[nmol/l]	0.89 ± 0.04	- -	19.55 ± 0.40
	bias / imprecision	[%]	-0.1 / 4.1	- -	2.4 / 2.1
Estradiol	Sample Identifier:		BCR-576	BCR-577	BCR-578
	certified value ±SD	[nmol/l]	0.114 ± 0.005	0.69 ± 0.04	1.34 ± 0.07
	found mean ±SD	[nmol/l]	0.120 ± 0.005	0.74 ± 0.03	1.35 ± 0.08
	bias / imprecision	[%]	4.9 / 4.7	6.9 / 7.6	0.4 / 4.2
Cortisol	Sample Identifier:		ERM-DA192	- -	ERM-DA193
	certified value ±SD	[nmol/l]	273 ± 6	- -	763 ± 14
	found mean ±SD	[nmol/l]	293 ± 4	- -	814 ± 21
	bias / imprecision	[%]	7.5 / 1.4	- -	6.7 / 2.5
Progesterone	Sample Identifier:		ERM DA-347	- -	BCR-348R
	certified value ±SD	[nmol/l]	10.1 ±0.2	- -	26.9 ± 1.2
	found mean ±SD	[nmol/l]	11.3 ± 0.1	- -	30.0 ± 0.4
	bias / imprecision	[%]	11.5 / 1.1	- -	11.6 / 1.4

Table S7: Mean bias and z-values from taking part in the UK NEQAS steroids proficiency testing scheme from distribution 470 to 493 (12/2019–01/2022) and average CV and n of the group mean used as target value for 17-OH-progesterone (17P), androstenedione (ANDRO), aldosterone (ALDO), cortisol (CL), dehydroepiandrosterone (DHEA), dehydroepiandrosterone sulfate (DHEAS), estradiol (E2), progesterone (PROG), and testosterone (TES).

	**Risch laboratory**						**Group mean average statistics**	
	**Bias**			***Z*-score**				
**Parameter**	**Mean [%]**	**SD [%]**	**n**	**Mean**	**SD**	**n**	**CV [%]**	**n**
CL, serum	4.5	5.2	80	0.7	0.8	80	7.3	16
CL, urine	4.9	10.4	52	0.4	1.1	52	18.5	49
17P	-1.7	5.7	80	-0.1	0.4	80	12.7	49
PROG, *ALTM*	-0.4	18.9	80	0.3	0.7	80	14.1	238
PROG, *MS*	9.8	8.1	78	1.2	1.2	19	13.4	3
E2, *ALTM*	-9.5	15.4	77	-0.5	0.9	77	14.7	219
E2, *MS*	2.7	8.4	77	0.4	0.8	77	10.6	13
E2-high, *ALTM*	-1.5	9.2	76	-0.2	1.0	76	10.6	103
E2-high, *MS*	4.6	5.3	79	0.5	5.3	21	13.0	4
TES, female	1.1	4.8	72	0.2	0.6	72	8.8	53
TES, male	0.8	3.8	72	0.1	0.6	72	6.5	38
ANDRO	-4.9	5.2	80	-0.5	0.6	80	9.5	49
DHEAS	3.8	4.2	80	0.5	0.5	80	7.7	20
ALDO	-2.3	17.6	62	-0.1	1.0	62	17.5	17

ALTM all methods trimmed mean as target, *MS* LC-MS group mean as target

Table S8: Mean recovery at three different spike concentration levels (n=3) for 11-deoxycorticosterone (11-DOC), 11-deoxycortisol (11-DF), 17-OH-progesterone (17P), 21-deoxycortisol (21-DF), androstenedione (ANDRO), aldosterone (ALDO), corticosterone (CC), cortisol (CL), cortisone (CN), dehydroepiandrosterone (DHEA), dehydroepiandrosterone sulfate (DHEAS), dihydrotestosterone (DHT), estradiol (E2), progesterone (PROG), and testosterone (TES).

	**Low level**			**Medium level**			**High level**		
**Analyte**	**Spike [nmol/L]**	**Recovery [%]**	**SD [%]**	**Spike [nmol/L]**	**Recovery [%]**	**SD [%]**	**Spike [nmol/L]**	**Recovery [%]**	**SD [%]**
11-DOC	0.30	92.6	7.2	0.61	91.1	4.5	2.42	89.5	3.6
17P	2.27	99.7	1.1	4.54	99.0	1.1	18.16	97.6	2.2
ANDRO	2.62	98.9	3.5	5.24	99.0	3.4	20.95	99.4	2.0
DHEA	34.7	96.1	1.9	69.3	96.3	2.2	277.4	94.1	2.4
DHT	0.344	94.0	7.6	0.689	94.4	6.5	2.754	95.7	5.6
PROG	8.0	106.7	2.4	15.9	106.0	5.0	63.6	103.5	2.9
TES	0.87	104.1	7.6	1.73	106.5	5.4	6.93	105.3	1.5
21-DF	0.29	96.1	4.1	0.58	95.2	5.9	2.31	90.7	1.3
11-DF	1.44	120.8	4.4	2.89	118.8	4.1	11.55	107.7	4.8
CC	11.5	106.4	2.9	23.1	105.1	4.0	92.4	97.7	4.5
DHEAS	1916	105.0	3.4	3832	103.7	2.9	15326	90.3	2.9
E2	0.092	112.8	3.5	0.184	112.2	3.3	0.734	110.5	3.0
CL	207	99.9	3.2	414	97.5	2.8	828	93.0	2.8
CN	13.9	87.7	9.2	27.7	87.5	6.8	55.5	88.8	4.3
ALDO	0.277	108.6	9.7	0.555	106.6	3.5	2.220	107.0	2.2

Table S9: Glycerophospholipid compounds monitored during optimization of sample preparation and their respective LC-MS/MS parameters.

Compound	Mass	Q1	Q3	DP	CE
	g/mol	m/z	m/z	V	V
16:0 Lyso-PC	495.6	496.5	184.1	85	40
18:1 Lyso-PC	523.7	522.5	184.1	85	40
18:0 Lyso-PC	521.7	524.5	184.1	80	40
12:0 PC	621.8	622.5	184.1	90	40
16:0 PC	734.0	734.7	184.1	90	40
16:0 18:2 PC	758.1	758.7	184.1	100	40
16:0 18:1 PC	760.1	760.5	184.1	105	40
18:0 PC	790.1	790.8	184.1	100	40
20:0 PC	846.3	846.8	184.1	90	40

Table S10: Matrix effect (ME), extraction recovery (ER) and process efficiency (PE) and according standard deviations (SD) as determined in the pre-post spiking experiment at two concentration levels of 11-deoxycorticosterone (11-DOC), 11-deoxycortisol (11-DF), 17-OH-progesterone (17P), 21-deoxycortisol (21-DF), androstenedione (ANDRO), aldosterone (ALDO), corticosterone (CC), cortisol (CL), cortisone (CN), dehydroepiandrosterone (DHEA), dehydroepiandrosterone sulfate (DHEAS), dihydrotestosterone (DHT), estradiol (E2), progesterone (PROG), and testosterone (TES). Calculated based on Analyte Peak Area and on Area Ratio (n = 6). For the extraction recovery post-spiked IS peak area was used for normalization correcting only for the instrumental part of the analysis process.

		Analyte Peak Area						Area Ratio					
**Analyte**	**Spike Level**	**ME**	**SD**	**ER**	**SD**	**PE**	**SD**	**IS norm. ME**	**SD**	**Post IS norm. ER**	**SD**	**IS norm. PE**	**SD**
		**[%]**	**[%]**	**[%]**	**[%]**	**[%]**	**[%]**	**[%]**	**[%]**	**[%]**	**[%]**	**[%]**	**[%]**
11-DOC	Low	82.4	2.8	83.1	4.8	63.2	2.1	102.4	4.9	85.3	4.5	100.6	2.7
	High	84.5	2.2	74.6	1.9	62.1	2.7	104.9	2.9	78.6	3.0	98.8	1.4
17P	Low	65.2	5.3	99.1	2.9	64.3	4.0	98.7	6.1	97.9	2.2	99.7	3.0
	High	65.4	3.9	95.7	2.1	62.3	3.9	99.0	3.0	97.2	4.9	96.7	2.7
ANDRO	Low	74.7	3.9	96.4	3.5	70.5	5.0	99.7	7.0	100.0	3.3	98.5	6.2
	High	73.4	2.7	92.8	2.1	67.5	2.6	97.9	2.6	95.7	4.3	94.3	0.9
DHEA	Low	61.2	4.9	96.6	6.8	58.8	4.0	100.1	5.3	94.7	7.6	103.0	6.4
	High	66.0	5.2	92.0	2.7	60.5	4.2	107.8	4.2	93.7	5.0	105.7	3.2
DHT	Low	55.1	5.4	95.8	2.1	51.8	5.3	88.4	5.3	91.1	2.8	90.7	7.0
	High	55.1	3.5	92.7	2.8	50.9	3.8	88.5	2.2	95.1	4.9	89.1	1.7
PROG	Low	99.4	7.8	91.2	2.2	88.3	4.1	101.4	6.3	92.0	1.8	100.7	1.6
	High	97.3	4.3	87.3	3.5	84.3	3.0	99.3	2.8	90.2	3.7	96.3	2.1
TES	Low	84.6	19.7	97.5	0.8	69.6	17.0	100.4	26.7	98.9	1.6	86.6	22.5
	High	81.7	4.1	96.0	1.4	76.0	6.4	96.4	4.0	98.9	4.2	94.1	6.4
21-DF	Low	45.8	4.9	98.2	3.9	44.8	3.9	97.8	5.2	101.7	5.4	96.6	3.2
	High	45.6	3.0	98.8	1.1	45.1	3.2	97.7	4.4	101.8	4.5	97.3	3.9
11-DF	Low	68.5	2.9	96.5	2.7	64.6	3.0	98.7	3.8	95.1	2.6	101.0	2.1
	High	68.3	3.1	90.7	1.5	61.5	3.1	98.4	2.2	93.2	4.0	96.1	1.6
CC	Low	67.6	6.6	95.0	4.2	60.1	3.4	94.9	10.2	94.7	7.4	102.8	4.3
	High	69.0	3.2	83.0	2.2	55.2	3.9	96.6	1.7	85.7	2.9	94.3	1.7
DHEAS	Low	119.0	18.8	92.6	2.9	93.9	10.9	125.6	21.1	94.2	3.2	118.5	12.8
	High	98.9	4.0	85.7	2.0	81.0	1.6	104.3	4.8	91.1	3.5	102.4	1.0
E2	Low	100.6	1.2	85.9	0.4	86.2	0.7	96.2	1.9	86.1	2.1	96.5	0.6
	High	100.9	1.4	85.9	1.7	86.6	1.2	96.5	2.1	89.1	2.7	97.0	1.2
CL	Low	71.4	15.2	97.7	1.6	58.5	6.4	93.1	20.0	97.4	3.7	83.8	8.4
	High	75.7	1.0	93.3	1.2	67.0	2.0	98.8	2.5	97.2	2.7	96.1	2.7
CN	Low	70.6	19.8	97.8	1.4	53.0	13.5	85.9	23.4	97.0	3.7	68.9	17.0
	High	78.6	0.9	92.7	1.3	67.4	2.7	95.9	2.0	95.5	2.7	87.5	3.2
ALDO	Low	60.4	3.0	88.5	2.3	49.4	2.8	109.8	7.3	87.2	2.7	106.3	7.0
	High	55.4	2.7	82.0	2.7	44.8	3.6	100.6	2.8	84.1	3.6	96.1	3.0

Table S11: Results from screening steroid compounds in pure solution as possible isobaric interferents. The retention time (RT) of each compound is compared to the RTs of isobaric analytes and all ion transitions that produced a signal are listed.

Compound	Structure	M [g/mol]	RT [min]	Isobar of analyte (RT [min])	Found in ion transitions [m/z]
Trenbolone	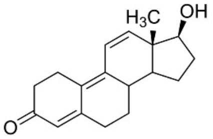	270.37	3.60	E2 (2.68)§	271→145/183 (−)<br>271→199 (+)‡<br>269→145 (−)‡
Norandrostenedione	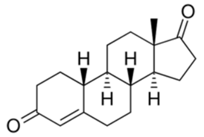	272.37	4.20	E2 (2.68)	271→145/183 (−)<br>273→109 (+)‡
17-alpha-estradiol	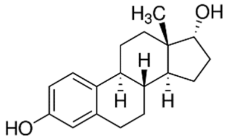	272.38	2.63	E2 (2.68)	271→145/183
Estrone	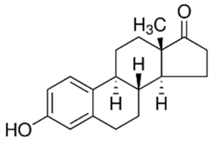	270.37	3.50	E2 (2.68)§	271→145/183<br>269→145‡
Estriol	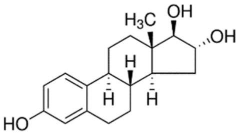	288.38	1.30	DHEA (3.65)<br>TES (3.84)	289→213/253
Epitestosterone	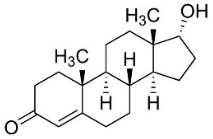	288.42	3.75	DHEA (3.65)<br>TES (3.84)	289→97/109<br>289→213/253
Etiocholanolone	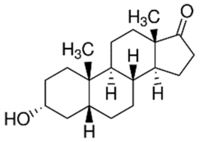	290.44	3.95	DHT (4.10)	291 → 255/159<br>273 → 255‡
Epiandrosterone	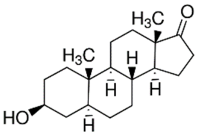	290.44	3.90	DHT	291→255/159<br>273→255/161/147‡
Androsterone	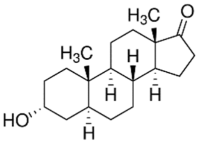	290.44	3.90	DHT	291→255/159<br>273→255/161/147‡
Testosterone acetate	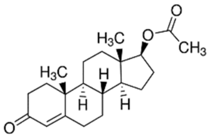	330.46	5.15	17P (3.84)<br>11-DOC (4.35)	331 → 97/109
Prednisone	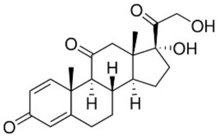	358.43	2.25	CN (2.35) §<br>ALDO (2.75)§	

Compound	Structure	M [g/mol]	RT [min]	Isobar of analyte (RT [min])	Found in ion transitions [m/z]
Prednisolone	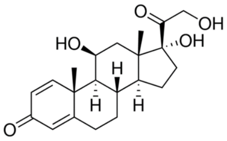	360.44	2.05	CL (2.10)§ CN (2.35) ALDO (2.75)	
18-OH-Corticosterone	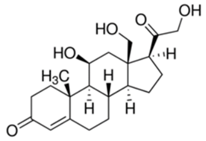	362.46	2.25	CL (2.10)	363→269 (+)‡ no signal in negative ion traces of CL
Dexamethasone	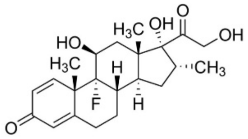	396.46	2.40	21-DF-d8(2.45)†	355→319 393→147‡

§ M+2 isotope as potential isobaric interference (e.g., 13C2 isotope)
† Isobaric interference caused by in-source fragmentation
‡ Specific ion transitions of the respective compound, not included in the assay
(+), (−) positive or negative ionization

Table S12: Accuracy in quality control samples of 11-deoxycorticosterone (11-DOC), 11-deoxycortisol (11-DF), 17-OH-progesterone (17P), 21-deoxycortisol (21-DF), androstenedione (ANDRO), aldosterone (ALDO), corticosterone (CC), cortisol (CL), cortisone (CN), dehydroepiandrosterone (DHEA), dehydroepiandrosterone sulfate (DHEAS), dihydrotestosterone (DHT), estradiol (E2), progesterone (PROG), and testosterone (TES) spiked with a mix of 13 potential steroidal interference compounds at concentration of 100 ng/mL or 1000 ng/mL. Analyte results ±15% off target are bolded.

Analyte	QC-I		QC-II		QC-III	
	QN [%]	QL [%]	QN [%]	QL [%]	QN [%]	QL [%]
11-DOC	101.0	99.6	99.6	99.8	101.0	101.0
17P	99.3	99.5	98.1	97.2	93.5	93.9
**ANDRO**	**249.0**	**250.0**	**127.0**	**128.0**	96.5	95.5
**DHEA**	**81.8**	**44200.0**	91.1	**7690.0**	97.6	93.0
DHT	103.0	95.0	98.1	109.0	96.9	93.7
PROG	98.2	97.9	99.7	100.0	95.9	94.1
**TES**	**138.0**	**13700.0**	90.9	94.1	95.8	96.7
**21-DF**	**58.6**	**67.7**	**49.8**	**52.5**	**46.5**	**45.5**
11-DF	98.2	98.0	85.6	86.0	102.0	101.0
CC	101.0	93.8	97.4	96.3	96.0	97.2
DHEAS	96.7	99.7	100.0	101.0	98.2	93.9
**E2**	**180000.0**	**59100.0**	**34000.0**	**11700.0**	**6000.0**	**2080.0**
CL	106.0	101.0	103.0	101.0	103.0	105.0
CN	103.0	96.0	98.2	90.9	96.7	98.0
ALDO	109.0	98.1	98.5	98.4	104.0	104.0

Table S13: Materials—consumables.

Type	Material	Producer	Order Nr.
Pipette tips	Combitips advanced (0.5 mL, 5.0 mL und 10 mL)	Eppendorf	0030089421<br>0030089456<br>0030089464
Pipette tips	Eppendorf epT.I.P.S.® (50–1000 µL, 2–200 µL, 100–5000 µL)	Eppendorf	0030075234<br>0030075250<br>0030075293
Tubes	Eppendorf tubes 3810X 1.5 mL, colorless, PP	Eppendorf	0030125.150
Tubes	Eppendorf Safe-Lock tubes 2.0 mL, colorless, PP	Eppendorf	0030 123.344
Tubes	Sample tubes 11.5 mL, 100 × 15.7 mm, PS	Sarstedt	55.466
LC vials	2 mL crimp-snap vials, glass, brown, 2 mL	WICOM	WIC42730
Snap caps	PP, FEP/butylgum septum	WICOM	WIC44760/R
Micro insert	300 µL, glass	WICOM	WIC47000
Phospholipid removal filter cartridges	HybridSPE Phospholipid Cartridge, 30mg bed weight, 1 mL volume	Supelco / Sigma Aldrich	55261-U (100 pcs)

Table S14: Materials—ISO17034 certified reference materials as solutions.

Analyte	Vendor	Identifier	Concentration	Solvent
Aldosterone	Cerilliant	A-096	100 µg/ml	Acetonitrile
Androstenedione	Cerilliant	A-075	1 mg/ml	Acetonitrile
Corticosterone	Cerilliant	C-117	1 mg/ml	Methanol
Cortisol	Cerilliant	C-106	1 mg/ml	Methanol
Cortisone	Cerilliant	C-130	100 µg/ml	Methanol
11-Deoxycorticosterone	Cerilliant	D-061	100 µg/ml	Methanol
11-Deoxycortisol	Cerilliant	D-061	1 mg/ml	Methanol
21-Deoxycortisol	Cerilliant	D-062	100 µg/ml	Methanol
DHEA	Cerilliant	D-063	1 mg/ml	Methanol
DHEAS	Cerilliant	D-065	1 mg/ml	Methanol
Dihydrotestosterone	Cerilliant	D-073	1 mg/ml	Methanol
17α-Hydroxyprogesterone	Cerilliant	H-085	1 mg/ml	Methanol
17β-Estradiol	Cerilliant	E-060	1 mg/ml	Acetonitrile
Progesterone	Cerilliant	P-069	1 mg/ml	Acetonitrile
Testosterone	Cerilliant	T-037	1 mg/ml	Acetonitrile

Table S15: Substances purchased for interference checks.

Analyte	Vendor	Identifier	Properties
Dexamethasone	Cerilliant	D-085	1 mg/mL solution in methanol
18-Hydroxycorticosterone	Cerilliant	H-106	100 µg/mL in 90 % acetonitrile
Trenbolone	Cerilliant	T-043	1 mg/mL in acetonitrile
Estrone	Cerilliant	E-075	1 mg/mL in methanol
Estriol	Cerilliant	E-074	1 mg/mL in methanol
Epitestosterone	Cerilliant	E-058	1 mg/mL in acetonitrile
Etiocholanone	Sigma Aldrich	32833	100 µg/mL in acetonitrile
Testosterone acetate	Sigma Aldrich	32676	100 µg/mL in acetonitrile
Norandrostenedione	LGC	LGCAMP0660.01-11	1 mg/mL in acetonitrile
17α-estradiol	Sigma Aldrich	E8750	Neat substance
Epiandrosterone	LGC	CDX-00005120-250	Neat substance
Prednisolone	Sigma Aldrich	46656-250mg	Neat substance
Prednisone	Sigma Aldrich	P2900000	Neat substance

Table S16: Target spiking concentrations for 11-deoxycorticosterone (11-DOC), 11-deoxycortisol (11-DF), 17-OH-progesterone (17P), 21-deoxycortisol (21-DF), androstenedione (ANDRO), aldosterone (ALDO), corticosterone (CC), cortisol (CL), cortisone (CN), dehydroepiandrosterone (DHEA), dehydroepiandrosterone sulfate (DHEAS), dihydrotestosterone (DHT), estradiol (E2), progesterone (PROG), and testosterone (TES). of samples used for the determination of recovery and matrix effect.

	Recovery (RSS)			Matrix effects (ASS)	
	Low [nmol/L]	Medium [nmol/L]	High [nmol/L]	Low [nmol/L]	High [nmol/L]
11-DOC	0.303	0.605	2.421	0.25	2.52
11-DF	1.44	2.89	11.55	1.20	12.0
17P	2.27	4.54	18.16	1.26	12.6
21-DF	0.29	0.58	2.31	3.61	36.1
ALDO	0.277	0.555	2.220	2.31	23.12
ANDRO	2.62	5.24	20.95	1.16	11.6
CC	11.55	23.09	92.36	4.81	48.1
CL	207	414	828	34.5	345
CN	13.9	27.7	55.5	4.6	46.2
DHEA	34.7	69.3	277.4	14.4	144.4
DHEAS	1916	3832	15326	426	4257
DHT	0.344	0.689	2.754	0.57	5.74
E2	0.092	0.184	0.734	0.31	3.06
PROG	7.95	15.90	63.60	2.12	21.2
TES	0.87	1.73	6.93	0.58	5.78

Table S17: Spiking scheme for the preparation of sample sets for assessment of matrix effects and extraction recovery.

Spike	Set A	Set B	Set C	Set D	Set E
	**"blank-matrix" [µL]**	**"post-post" [µL]**	**"pre-post" [µL]**	**"pre-pre" [µL]**	**"blank-spike" [µL]**
Pre					
Sample	0	100	100	100	100
PPT	0	200	200	200	200
ISS	20	0	0	20	0
ASS	20	0	20	20	0
Blank	300	0	0	0	40
Post					
Eluate	-	150	160	170	170
ISS	-	10	10	0	0
ASS	-	10	0	0	0

Table S18: Details and performance data provided by the manufacturers of immunoassays which are currently in use by Dr. Risch laboratories for 17-OH-progesterone (17P), androstenedione (ANDRO), cortisol (CL), dehydroepiandrosterone (DHEA), dehydroepiandrosterone sulfate (DHEAS), estradiol (E2), progesterone (PROG), and testosterone (TES).

**Analytes**	**CL**	**DHEAS**	**E2**	**PROG**	**TES**	**ANDRO**	**17P**	**DHEA** [1]
Platform			Roche			Siemens	IDS	Demeditec
			Elecsys Cobas e411/e601/e602			Immulite	iSYS	---
Principle	ECLIA	ECLIA	ECLIA	ECLIA	ECLIA	CLIA	CLIA	ELISA
LOD [nmol/l]	1.5	5	0.018	0.087	0.087	n.a.	0.45	0.3
LLOQ [nmol/l]	3.0	81	0.092	0.636	0.416	1.1	0.9	1.0
ULOQ [nmol/l]	1750	27,100	11.010	191.0	52.0	35	48.5	104.2
with dilution (factor)	17,500 (1:10)	135,700 (1:5)	110.1 (1:10)	n.a.	n.a.	n.a.	97.0 (1:2)	n.a.
Repeatability CV %	1.6–1.7	1.5–3.2	1.1–8.5	1.9–5.3	1.2–8.9	6.2–15.1	1.2–5.3	6.4–8.2
Intermediate Prec. CV %	1.9–2.3	2.2–4.7	1.9–11.9	3.2–10.4	1.6–14.5	8.5–17.8	3.9–11.1	4.7–10.3

[1] External partner laboratory
